# Extensive genome analysis of *Coxiella burnetii* reveals limited evolution within genomic groups

**DOI:** 10.1186/s12864-019-5833-8

**Published:** 2019-06-05

**Authors:** Claudia M. Hemsley, Paul A. O’Neill, Angela Essex-Lopresti, Isobel H. Norville, Tim P. Atkins, Richard W. Titball

**Affiliations:** 1 College of Life and Environmental Sciences – Biosciences, University of Exeter, Exeter, UK; 2 Defence Science and Technology Laboratory, Porton Down, Salisbury, UK

**Keywords:** *Coxiella burnetii*, Whole Genome Sequencing, Genotyping, Pan-Genome Analysis, Patho-adaptation

## Abstract

**Background:**

*Coxiella burnetii* is a zoonotic pathogen that resides in wild and domesticated animals across the globe and causes a febrile illness, Q fever, in humans. An improved understanding of the genetic diversity of *C. burnetii* is essential for the development of diagnostics, vaccines and therapeutics, but genotyping data is lacking from many parts of the world. Sporadic outbreaks of Q fever have occurred in the United Kingdom, but the local genetic make-up of *C. burnetii* has not been studied in detail.

**Results:**

Here, we report whole genome data for nine *C. burnetii* sequences obtained in the UK. All four genomes of *C. burnetii* from cattle, as well as one sheep sample, belonged to Multi-spacer sequence type (MST) 20, whereas the goat samples were MST33 (three genomes) and MST32 (one genome), two genotypes that have not been described to be present in the UK to date. We established the phylogenetic relationship between the UK genomes and 67 publically available genomes based on single nucleotide polymorphisms (SNPs) in the core genome, which confirmed tight clustering of strains within genomic groups, but also indicated that sub-groups exist within those groups. Variation is mainly achieved through SNPs, many of which are non-synonymous, thereby confirming that evolution of *C. burnetii* is based on modification of existing genes. Finally, we discovered genomic-group specific genome content, which supports a model of clonal expansion of previously established genotypes, with large scale dissemination of some of these genotypes across continents being observed.

**Conclusions:**

The genetic make-up of *C. burnetii* in the UK is similar to the one in neighboring European countries. As a species, *C. burnetii* has been considered a clonal pathogen with low genetic diversity at the nucleotide level. Here, we present evidence for significant variation at the protein level between isolates of different genomic groups, which mainly affects secreted and membrane-associated proteins. Our results thereby increase our understanding of the global genetic diversity of *C. burnetii* and provide new insights into the evolution of this emerging zoonotic pathogen.

**Electronic supplementary material:**

The online version of this article (10.1186/s12864-019-5833-8) contains supplementary material, which is available to authorized users.

## Background

*Coxiella burnetii* is an obligate intracellular pathogen and the etiological agent of Q-fever, a zoonotic disease of humans which has been reported from almost every country worldwide [1]. The clinical presentation is pleomorphic and includes severe forms associated with a poor prognosis [2]. The bacterium can be isolated from a wide range of wild and domestic animals, including cattle, sheep, goats, cats, and dogs [3]. Some of these may serve as reservoirs for the bacterium. In many of these animal hosts, the infection is chronic and virtually asymptomatic. The animal hosts most frequently implicated as sources of human infection are domesticated livestock such as sheep, goats and cattle [4].

An improved understanding of the genetic diversity of *C. burnetii* and its virulence mechanisms is essential for the development of diagnostics, vaccines and therapeutics. The genome sequence of the Nine Mile I (NM-I) reference strain reveals a 1,995,275-bp chromosome and a 37,393-bp previously sequenced QpH1 plasmid [5]. Genome analysis has shown a high proportion of genes that are annotated as hypothetical proteins with no known function (719 genes = 33.7% of the genome) and also identified 83 pseudogenes suggesting that some genome reduction is underway [5]. Very few virulence-associated genes are annotated and virulence mechanisms of *C. burnetii* are still poorly understood. The lipopolysaccharide (LPS) was the first validated virulence factor [6]. Type I, II and IV secretion systems are also present in *C. burnetii* [5] and there is good evidence that the type IV secretion system (T4SS) plays a role in disease [7]. Interestingly, comparative genome analysis has revealed variations in the repertoire of the effectors secreted by the T4SS in strains with different genetic backgrounds [7–11], including plasmid encoded effectors [12]. There is also evidence of antigenic variation between *C. burnetii* isolates, which includes both the O-antigen of the lipopolysaccharide (LPS) as well as antigenic proteins [13]. Several studies using polyclonal and monoclonal antibodies revealed different binding patterns with LPS from different *C. burnetii* isolates [14, 15]. Strain-specific monoclonal antibodies were identified in cross-reactivity studies between isolates causing acute vs chronic disease [16, 17], but the genetic basis for this was not determined.

The diversity amongst *C. burnetii* isolates is not restricted to effector proteins and LPS biosynthesis, but extends to the broader genome content. Six genomic groups (GGs) have been proposed by restriction endonuclease digestion patterns [18], which have later been confirmed by Multiple-Locus Variable number tandem repeat Analysis (MLVA) [19] and Multispacer Sequence Typing (MST) [20]. GG I contains the NM-I reference strain and GG I isolates can be found across the globe [21, 22]. In contrast, GG II isolates have been mostly found in Europe and include the MST33 genotype that has been implicated in the largest Q fever epidemic in the Netherlands between 2007–2010 [23]. GG III is dominated by MST20, a genotype that is usually associated with cattle [24]. GG IV contains amongst others MST8, a genotype that has been linked to goats [24], and seems to harbor isolates with different metabolic requirements to other cultured strains, since many laboratories report failure of axenic culture of these isolates in ACCM-2 medium, which is tailored to the metabolic requirements of the Nine Mile strain [25, 26]. GG V contains a single genotype (MST21), which is endemic in Nova Scotia and surrounding parts of North America [22], whereas GG VI contains three rodent isolates obtained in Dugway, Utah, which are considered avirulent in humans [27, 28]. All other genomic groups contain isolates from cases of human disease [19]. In animal models, it has been shown that GG I isolates cause severe acute disease in guinea pigs and GG V isolates cause mild to moderate acute disease, whereas GG IV and VI isolates cause no acute disease at all [29]. However, a different guinea pig study showed that strain MSU_Goat_Q177 (Priscilla; GG IV) was as infectious as the NM-I strain in its ability to cause seroconversion and colonize the spleen, but only induced fever at a high infectious dose [6]. Mouse models have been used to compare a limited number of strains, which also showed that GG I isolates were most virulent [29, 30]. Two Belgian isolates have been studied in a BALB/c mouse model, which found similar colonization and clearance rates for the bovine (presumably GG III) isolate and NM-I, whereas the caprine isolate (GG II) showed a slower colonization rate in spleens, but was not completely cleared by 8 weeks post infection like the other two isolates [31]. Strain Idaho_Goat_Q195 from GG III has also been tested in guinea pigs and was found to be weakly virulent [32]. More comprehensive animal studies were performed in the middle of the last century [5, 33], but genotyping or genome data for most of these strains do not exist.

Whole genome sequences of 67 *C. burnetii* isolates were publically available at the time of submission. Out of the 55 described *C. burnetii* MST types, only 14 are represented by these sequences, leaving many genotypes without a sequenced representative. Most sequenced isolates are from Europe and North America. Only nine isolates from other continents have been sequenced, and these show some unique MST genotypes, most of which fall into GG IV [22], which suggest that the genetic diversity of *C. burnetii* worldwide may be even greater than currently described.

Limited data on the genetic make-up of *C. burnetii* in the UK exists. Only two entries of UK isolates have been made into the MVLA database [34], and no whole genome sequence data is available despite reports of Q fever in the UK as early as 1949 [35] and isolation of the infective agent from a human case and cow’s milk [36]. These UK isolates were reported to be more virulent than the Henzerling strain, a GG II isolate, in a guinea pig model [36]. 904 cases of acute Q fever were reported in England and Wales between 2000 and 2015, which included two recognized outbreaks in 2002 and 2007 [37], and a large Q fever outbreak in Scotland with 110 cases was recorded in 2006 [38]. Prior to that, eight outbreaks in the United Kingdom were reported between 1980 and 1996 [4]. *C. burnetii* is endemic in UK dairy cattle herds, with a reported seroprevalence of up to 12.5% in large dairy herds in Northern Ireland [39]. Tests on bulk tank milk from dairy cattle herds in England and Wales showed an overall herd prevalence of between 22% and 80% [40–42]. Seroprevalence for sheep (12.3% vs 9%) and goat (9.3% vs 26%) herds are reported for Northern Ireland and Great Britain, respectively [39, 43]. Wild rodents (up to 53% of rats), foxes (41.2%) and domestic cats (61.5%) in the UK also tested positive for *Coxiella* antibodies [44, 45].

In this study, we provide nine *C. burnetii* draft genomes obtained in the United Kingdom, all of which were from abortion material from ruminants. We present a new method to obtain *C. burnetii* DNA from complex samples such as placentas, and provide a comparative analysis of 67 available *C. burnetii* genomes. Our results provide new insight into the genomic diversity of *C. burnetii* and suggest evolution by clonal expansion, with very little variability being observed between isolates within a genomic group.

## Results

### Properties of *C. burnetii* genome sequences obtained from the UK

Nine samples for sequencing were obtained from abortion material from UK ruminants. *C. burnetii* gDNA was obtained from placental material by immunoaffinity capture, whereby an anti-*Coxiella* antibody coupled to magnetic beads was used to selectively isolate bacteria, allowing the subsequent isolation of *Coxiella* DNA (see Methods). Sufficient quantities of *Coxiella* DNA were extracted from nine placenta samples (4x cow, 4x goat, and 1x sheep). Other samples with a lower *C. burnetii* content (assessed using qPCR as < 1x10^5^ GE/ml) did not result in DNA of sufficient quantity and quality for downstream applications such as whole genome amplification [21, 46] and sequencing.

The properties of the nine *C. burnetii* draft genomes from the UK are described in Table 1. No large-scale deletions or insertions were detected compared to the NM-I reference genome, but several smaller deletions resulting in the complete or partial disruption of open reading frames were found, particularly in the genomes derived from goat placentas (Additional file 1: Table S1). The number and effects of all single nucleotide polymorphisms (SNPs) in the UK genomes compared to the NM-I genome as a reference was also analyzed and the results are summarized in Additional file 2: Table S2. The genomes from goats had the greatest total number of variants (2,762 - 2787) compared to genomes derived from cow and sheep samples (2,026 - 2,113). Two thirds of all variants were found to occur within coding sequences, with between 97 and 151 of these having a severe, high impact on the function of the encoded gene products.

**Table 1:** Statistics for sequencing, assembly, and annotation for the nine *C. burnetii* genomes sequenced in this study. The annotation data for strain Nine Mile RSA493 and corresponding QpH1 plasmid is included for comparison. Note that Cb_D1 was sequenced at 250-bp read length, whereas all other strains were sequenced as 150-bp reads.

Name	Source	QC passed reads	Mapped reads (%)^a^	Coverage	# contigs	Genome size (bp)	% GC	Predicted # CDS RAST/Prokka
Cb_D1	Cow placenta	2,826,398	563,469 (19.94%)	77.51	42	2,000,727	42.5	2,225/2,017
Q532	Cow placenta	2,046,051	1,800,928 (88.02%)	106.82	38	2,001,903	42.5	2,223/2,021
Q545	Cow placenta	2,351,449	2,187,509 (93.03%)	131.80	37	2,003,604	42.5	2,228/2,021
Q556	Cow placenta	2,150,728	1,170,716 (54.43%)	69.23	42	2,004,954	42.5	2,234/2,024
Q559	Sheep placenta	2,260,768	1,441,913 (63.78%)	88.30	39	2,004,244	42.5	2,230/2,023
Q540	Goat placenta	2,823,227	2,778,111 (98.40%)	165.78	111	2,010,957	42.5	2,306/2,036
Cb_D2	Goat placenta	2,219,644	2,104,345 (94.81%)	141.98	111	1,991,633	42.5	2,245/2,018
Cb_D8	Goat placenta	2,170,264	2,098,022 (96.67%)	140.28	113	1,993,660	42.5	2,257/2,019
Cb_D10	Goat placenta	1,162,812	1,127,480 (96.96%)	72.77	113	1,994,548	42.5	2,259/2,022
RSA493 + QpH1	Tick	n.a.	n.a.	n.a.	2	2,032,674	42.6	2,217/2,056

^a^ against Nine Mile RSA493 genome (AE016828.2 and AE016829.1 concatenated)

### Genotyping and phylogenetic relationship of 76 sequenced *C. burnetii* isolates

**Fig. 1:**
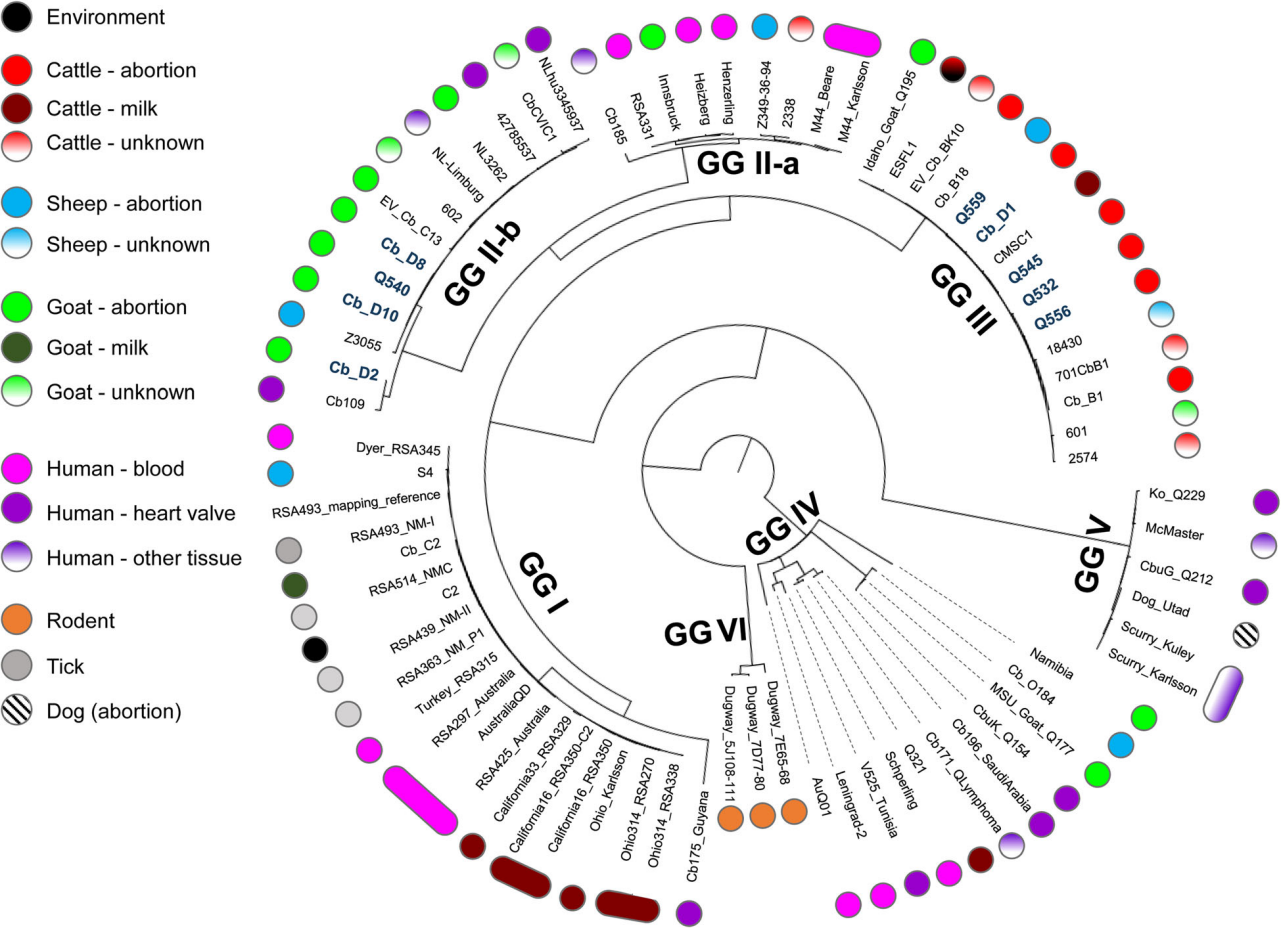
ParSNP tree of 76 *C. burnetii* isolates overlaid with associated metadata on source of isolation. The same SNP-based tree as seen in Additional file 5: Figure S1, is presented in a radial form; metadata is colour coded according to the legend shown next to the figure. The tree was rooted along the branch leading to GG IV (see Methods). The nine UK genomes are highlighted in bold in the Figure

Genome sequence data for the nine UK samples were analyzed together with 67 publically available *C. burnetii* genomes (see Additional file 3: Table S3 and Additional file 4: Data set S1-A for details). SNP data was used to establish the phylogenetic relationship between genome sequenced *C. burnetii* isolates using the Harvest suite tools. The included ParSNP aligner identifies SNPs within the aligned core genome, which then can be used to reconstruct the phylogeny. SNP densities were visualized using Gingr (Additional file 5: Figure S1). A radial view of the SNP-based phylogenetic tree is shown in Fig. 1, which revealed seven distinct phylogenetic clades. The UK cow and sheep samples clustered with other ruminant strains from Europe [47, 48] as well as the US [49]. Three of the four UK goat samples clustered with other European goat abortion isolates and human isolates that were implicated in the recent Q fever outbreak in the Netherlands [50], whereas the fourth UK goat sample (Cb_D2) clustered with a human heart valve isolate from Germany (Cb109) [51].

Next, *in silico* Multi-Spacer Sequence Typing (MST-typing) was performed on all genomes apart from passage variants (see Additional file 4: Data set S1-B). The goat samples Q540, Cb_D8, and Cb_D10 belonged to MST33, like the Dutch outbreak strains, whereas the remaining goat sample, Cb_D2, was MST32, like strain Cb109. All four cow samples and the sheep sample were MST20. The MST type of all previously genotyped isolates was confirmed by our method, except for strain Cb196_SaudiArabia, which returned MST4 in our analysis and strain Dugway 5J108–111, which returned a novel MST type. The two other Dugway isolates showed the same MST genotype as Dugway 5J108-111. Isolates Cb171_QLymphoma, Cb109, Q321, and Cb185 could not be properly assigned to a MST type due to poor sequence quality.

**Fig. 2:**
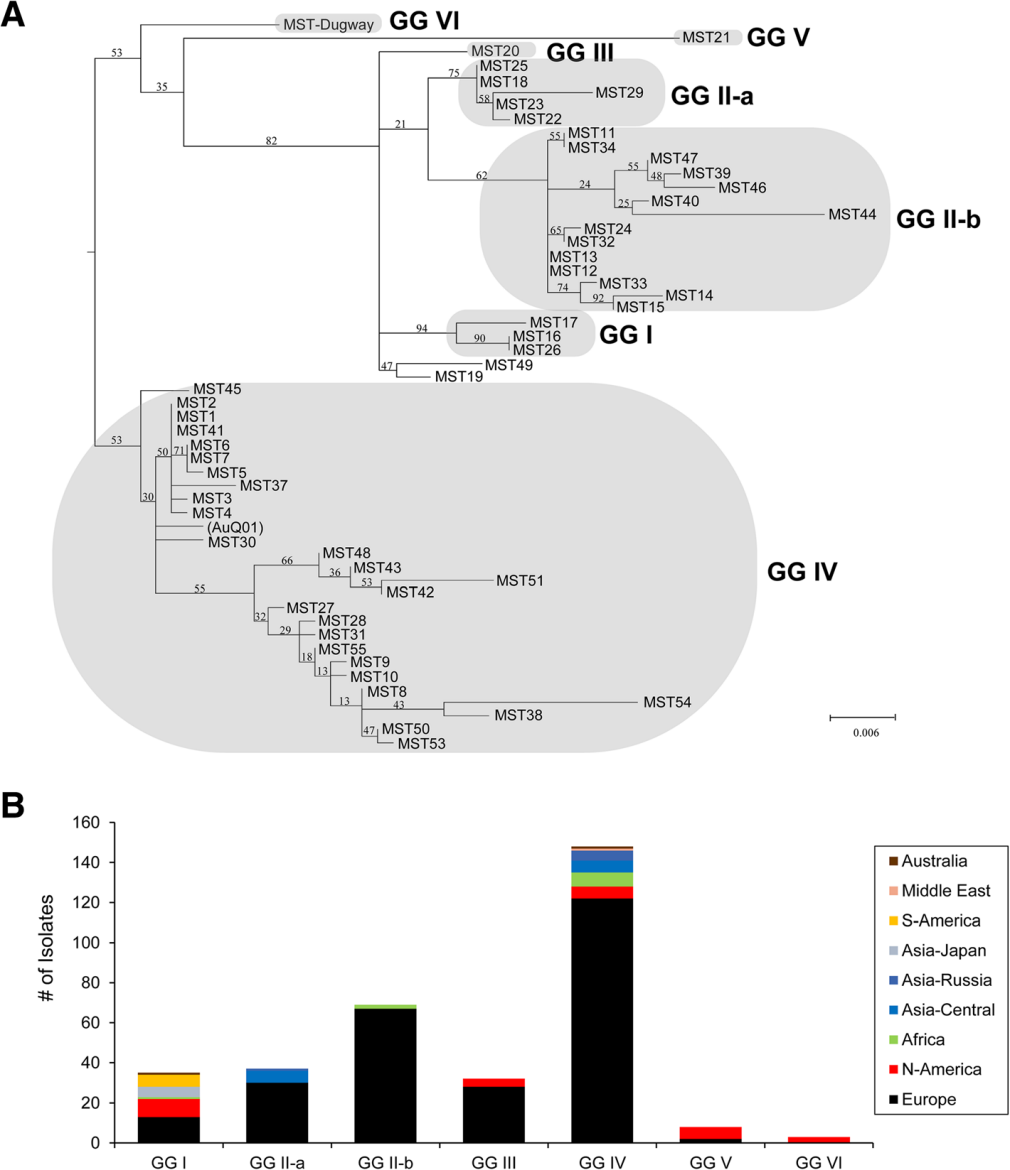
Analysis of MST genotype data of all *C. burnetii* isolates submitted to the MST database. **a** PhyML tree of all 55 known allele combinations. The suggested genomic groups highlighted are similar to Fig. 1 in Hornstra *et al.* [20]. The tree was rooted along the branch leading to GG IV (see Methods). **b** Number of isolates per genomic group with a described MST genotype ranked by their country of origin. Genotypes were assigned to a GG according to the tree shown in panel **a**)

A PhyML phylogenetic tree was constructed using the *in silico* MST alleles of any novel MST types and including previously published MST sequences. As seen in Fig. 2a, the MST tree supported the suggested placement of MST genotypes into the six genomic groups defined by Hendrix *et al*. [18]. MST19 and closely related genotype MST49 were originally assigned to GG III by Hornstra [20], but, using the analysis described here, they did not cluster well with the MST20 genotype. We also analyzed the metadata for each genotype in the MST database (n = 312) and plotted the number of isolates belonging to each genomic group according to their continental origin (Fig. 2b). This confirmed that most European isolates belong to GG I to IV, whereas GG V and VI are dominated by North American isolates. Isolates from other continents predominantly fall into GG IV. However, it has to be pointed out that European and North American isolates are over-represented in this database and more data from other parts of the world and from more variable isolation sources are required to confirm these trends.

Next, *in silico* plasmid typing and Acute Disease Antigen A (*adaA*) typing were performed. We found novel SNPs to which we assigned a version number (V2&3; see Additional file 6: Figure S2). The *adaA* genotypes observed (Additional file 6: Figure S2) were restricted to certain genomic groups: The two deletion types (Δ1 and Δ2.1) were only found in GG IV and GG V, respectively. The previously reported SNP genotype (SNP_orig_) was found in one subgroup of GG II which we have named GG II-a, and which included strains Cb185, RSA331, Innsbruck, M44, 2338, Z349-36/94, Henzerling, and Heizberg. Most draft genomes in the MST33-subgroup of GG II (here named GG II-b) did not produce an *in silico* PCR product due to a genomic rearrangements in the *adaA* region (data not shown), with the exception of the curated genome of strain Z3055, which only differed from the reference (GG I) *adaA* region by 24 SNPs. When the sequencing reads of samples Q540, Cb_D8, or Cb_D10 were mapped onto the complete Z3055 genome, no SNPs were detected in the *adaA* region, suggesting the same genomic configuration here termed SNP_V2_. The two MST32 genomes Cb109 and Cb_D2 also exhibited genomic rearrangements in the *adaA* region, which were slightly different compared to MST33 isolates, but with little or no sequence variation (0 and 2 SNPs in Cb_D2 and Cb109 compared to Z3055, respectively). GG III isolates all showed a SNP_V3_ genotype. A summary of all genotyping results can be seen in Additional file 7: Figure S3.

Lastly, subtyping of all 15 MST20 isolates based on 82 SNPs defined by Olivas *et al.* [49] was performed, which showed that all European MST20 belonged to sub-genotype GT_20.1, whereas three out of the four US isolates belonged to sub-genotype GT_20.2 and GT_20.3 (see Additional file 8: Figure S4). Interestingly, the five MST20 genomes obtained in the UK did not cluster together but interspersed with isolates from other parts of Europe or, in one case, from the USA.

### Genome comparisons and pan-genome analyses

**Fig. 3:**
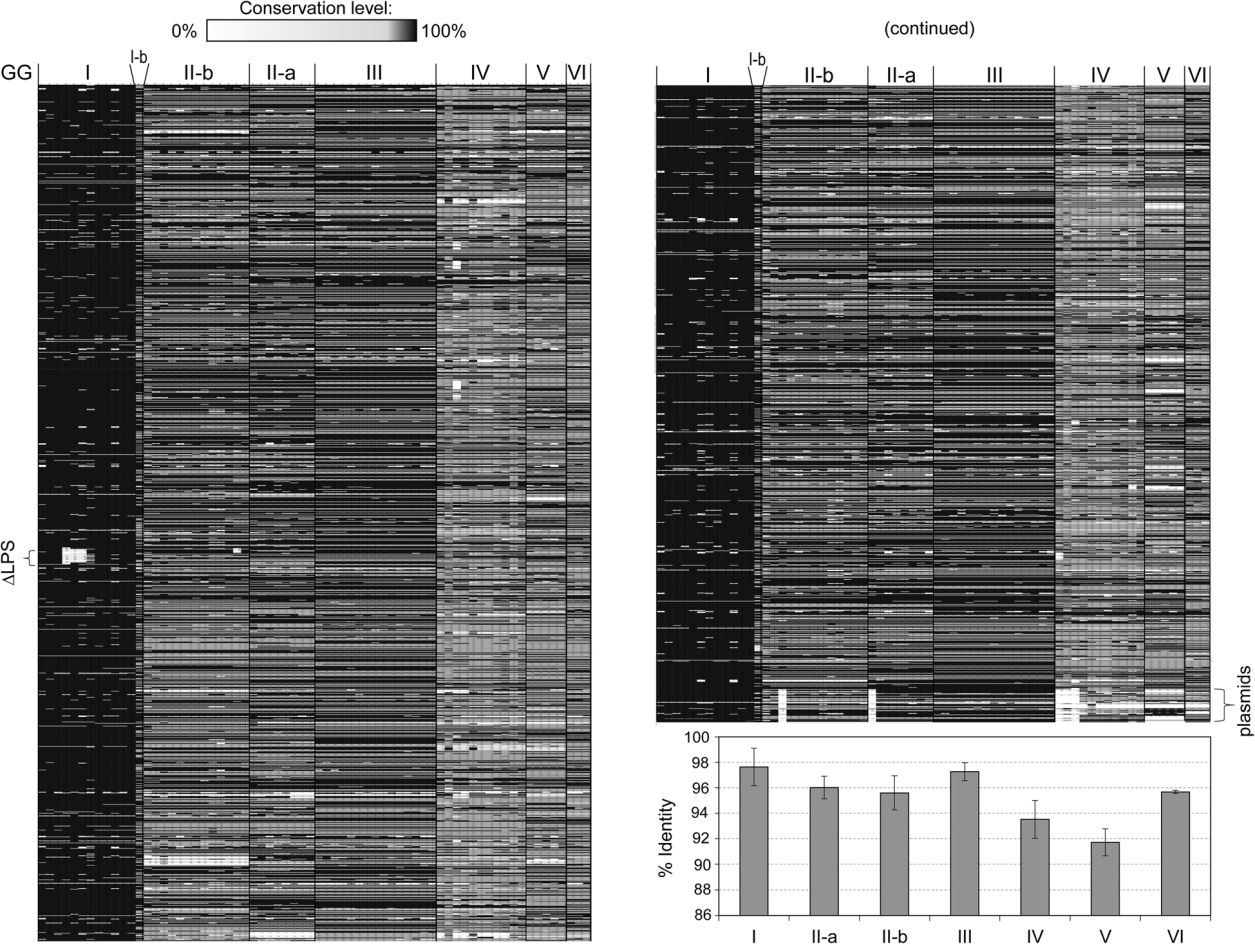
Heat maps of gene conservation levels across the available *C. burnetii* genomes compared to the NMI reference strain. Gene conservation data was obtained from SEED viewer (see Methods). Note that plasmid data is absent for strains Cb196_SaudiArabia, Cb175_Qlymphoma, Q321, Schperling, Z3055 and Cb185. The inset graph shows average sequence conservation levels for each genomic group with standard deviation. Genomic groups were assigned as seen in Fig.1. Cb175_Guyana was here labelled as GG I-b

First, we analyzed sequenced genomes for gene conservation compared to the NM-I strain. Each genomic group had a distinct pattern (Fig. 3) whereas strain Cb175_Guyana showed a unique pattern that suggested that this strain does not cluster with GG I isolates, as already seen in the SNP tree (Additional file 5: Figure S1). The strain-specific gene conservation levels ranged between 87.5% and 98.8% (Additional file 4: Data set S1-C), whereas the average conservation per genomic group ranked these (in decreasing order) as GG I > GG III > GG II > GG VI > GG IV > GG V (see inset graph in Fig. 3).

Next, a pan-genome analysis was performed to determine the core and accessory genomes of all sequenced *C. burnetii* isolates excluding passage variants. We first identified the least stringent condition that would allow for any miss-annotation to be tolerated without resulting in false positives (see Methods). Using 67 Prokka annotated genomes and a protein similarity threshold of 90%, the BPGA pipeline predicted 1311 core genes present in all genomes, whereas the Roary pipeline predicted 989 core genes and 318 soft-core genes that are present in 63-66 genomes (Additional file 9: Figure S5 and Additional file 4: Data set S1-D). Genomes with lower sequence quality (Cb171_QLymphoma, Cb109, Q321 and Cb185) exhibited larger numbers of unique and exclusively absent genes, suggesting some misclassification. In other genomes, the majority of “new” or “unique” genes were found to encode polymorphic variants of proteins due to frameshift and missense mutations, whereas most “absent” genes were found to contain SNPs that introduced a premature stop codon. This indicates that the pan genome results obtained do not report new genome content in the classic sense, but can be used to report pseudogenization events instead. The phylogenetic relationship based on the core and pan genome content, respectively, resulted in phylogenetic trees that clustered the strains according to the genomic groups assigned in Fig. 1, with a few exceptions of strains with lower sequence quality or missing plasmid sequences (Additional file 10: Figure S6).

Finally, we used the Panther Gene List analysis tool (see Methods) for functional classification to compare the functions encoded by the various parts of the genomes. We found that the core genome showed a slight but significant 1.25-fold enrichment in genes encoding proteins with “catalytic activity” as their molecular function, or “metabolic process” and “cellular process” in the Biological Process category (Additional file 11: Table S4). “Intracellular” in the Cellular Component category was also enriched 1.24-fold. In contrast, the accessory genome showed a depletion of genes belonging to these categories. No significant hits were obtained with unique genes as input (data not shown). It is noteworthy, however, that 93% of genes in the core genome could be assigned a UniprotID, whereas only 62% and 72% of genes in the accessory and unique genome, respectively, could be assigned to an ID.

### Genomic Group specific pan-genome analysis and pan-GWAS

**Fig. 4:**
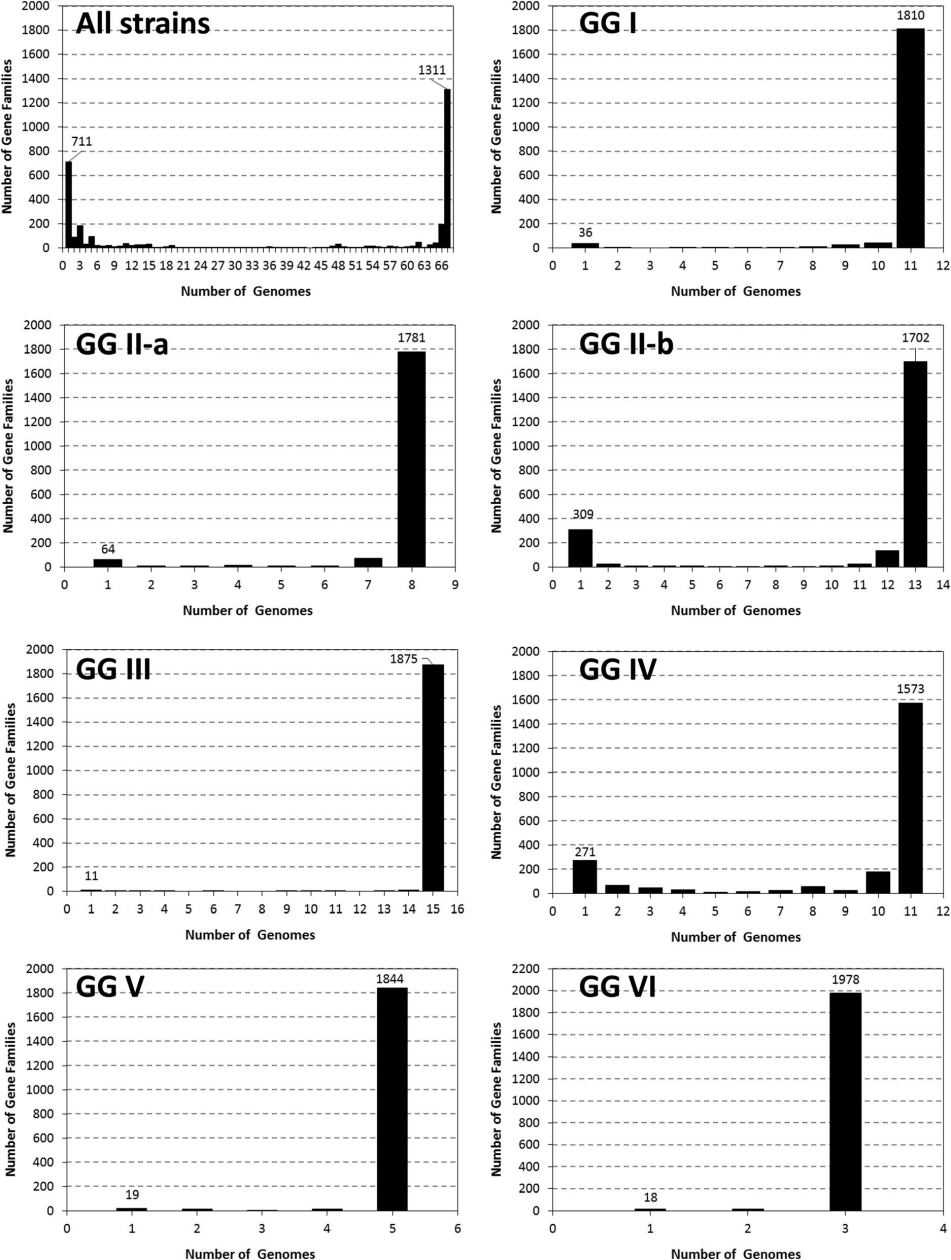
Gene frequency plots after BPGA pan-genome subset analysis using genomic group associations. Proteins annotated using PROKKA were used as input files. The protein similarity threshold for protein clustering was 90%. The bars furthest to the right in each graph represent conserved core-genes; the bars furthest to the left in each graph represent unique genes. The number of core genes is indicated within each figure

The genomic groups assigned in Fig. 1 were used for a subset analysis in BPGA. This revealed that each genomic group had a different proportion of genes in the core genome (Fig. 4), which coincided with the degree of clustering observed in the SNP tree. GG VI, containing the three Dugway strains, was the least variable subgroup, with 1978 genes being assigned to the core genome. Genomic groups III, I, II-a, and V also exhibited low diversity with 1781 to 1875 core genes. GG IV was the most diverse subgroup with only 1573 genes assigned to the core genome, whereas GG II-b exhibited most new genes (= polymorphic variants) per genome addition (Additional file 12: Figure S7).

We also identified genes with predicted functions that were unique for genomic groups (Table 2 and Additional file 4: Data set 1S-E for raw data). One aminotransferase family protein, and a Fic-domain protein were absent in GG I. The four genes that have been previously identified as being partially or completely deleted in the UK goat samples (Additional file 1: Table S1) as well as a hypothetical protein containing a mannan-binding (MVL family) domain were absent from all GG II-b isolates. A NudE/NUDIX family protein (CBU_0598), which has previously been reported to be absent in the Idaho_Goat strain [21], was absent in all members of GG III. The majority of the genes that were absent from GG V only were plasmid genes. Overall, a significant proportion of GG-specific genes encoded T4SS substrates, including nine that are annotated in RSA493, one immunogenic protein, and two additional possible substrates from other genomic groups.

**Table 2:** Genomic Group-specific genome content

Absent from	ID in RSA493	Function	Absent from	ID in RSA493	Function
GGIIa	CBU_0584	hypothetical protein	GGV	CBU_1158	7-dehydrocholesterol reductase
GGIIa	CBU_0945	membrane-assoc. protein	GGV	CBU_1308	phosphohydrolase; HD domain containing
GGIIa	CBU_0978	membrane-assoc. protein, T4SS substrate	GGV	CBU_1460*	hypothetical protein; T4SS substrate
GGIIa	**CBU_1209**	membrane-spanning protein	GGV	**CBU_1664**	CBS domain protein
GGIIa	CBU_1213	ankyrin repeat-containing protein; T4SS substrate	GGV	**CBU_1665**	hypothetical protein; T4SS substrate
GGIIa	CBU_1404	hypothetical protein	GGV	CBU_1788	DNA-binding protein, KilA-N
GGIIa	**CBU_1991**	toxin-antitoxin system antitoxin RelB	GGV	**CBU_1800**	membrane-spanning protein
GGIIa	**CBU_1992**	toxin-antitoxin system antitoxin RelE	GGV	**CBU_1801**	hypothetical protein
GGIIb	CBU_0880	hypothetical protein	GGV	**CBU_1802**	hypothetical protein
GGIIb	CBU_1100	hypothetical protein	GGV	**CBU_1803**	hypothetical protein
GGIIb	CBU_1103	lytic transglycosylase	GGV	**CBU_1804**	LuxR family transcriptional regulator
GGIIb	CBU_1111	membrane-bound lytic murein transglycosylase	GGV	**CBU_1805**	LuxR family transcriptional regulator
GGIIb	CBU_1112	GIY-YIG catalytic domain protein; endonuclease	GGV	**CBU_1806**	hypothetical protein
GGIII	CBU_0590	hypothetical protein; T4SS substrate	GGV	CBU_1895	hypothetical protein
GGIII	**CBU_0598**	ADP compounds hydrolase NudE	GGV	**CBUA0001**	helix-turn-helix domain containing protein
GGIII	CBU_0686	pyruvate dehydrogenase E1 subunit alpha	GGV	**CBUA0003**	cell filamentation protein
GGIII	CBU_1710	hypothetical protein	GGV	**CBUA0028**	RelE/ParE family toxin
GGIII	CBU_1723	protein-disulfide reductase DsbD	GGV	**CBUA0032**	3',5'-cyclic-nucleotide phosphodiesterase
GGIV	CBU_0777	hypothetical protein	GGV	**CBUA0033**	hypothetical protein
GGIV	CBU_0860	hypothetical protein	GGV	**CBUA0036**	chromosome partitioning protein
GGIV	CBU_1379a	hyp. protein; T4SS substrate	GGV	**CBUA0037**	ParA protein
GGIV	CBU_1618	hypothetical protein	GGV	**CBUA0038**	ParB protein
GGIV	CBU_2041	PAS domain S-box protein	GGV	**CBUA0039**	RepA protein
GGV	CBU_0007a	BrnT family toxin	GGV	**CBUA0039a**	hypothetical protein
GGV	CBU_0183	hyp. protein; T4SS substrate	GGVI	CBU_0793	hypothetical protein
GGV	CBU_0196	hypothetical protein	GGVI	CBU_1092	lipoprotein
GGV	**CBU_0562**	hypothetical ATPase	GGVI	CBU_1466	hypothetical protein
GGV	CBU_0705	hypothetical protein	GGVI	CBU_1822	SodC superoxide dismutase
GGV	CBU_0948	hypothetical protein	GGVI	CBU_1932	hypothetical protein
GGV	**CBU_0953**	amino acid permease	GGVI	**CBUA0024**	hypothetical protein

Proteins classed as absent in one GG only by BPGA subset analysis were searched for homologues in the RSA493 reference genome. Genes that have been also been shown to be group specific by Beare *et al*. [21] are highlighted in bold. The asterisk indicates an immunoreactive protein [65]

Finally, a Genome-Wide Association Study (GWAS)-like analysis of the pan genome was performed, using phenotypic traits such as country / continent of origin, host source, genomic group and MST type of the strain collection as queries (see Methods and Additional file 4: Data set S1-F for raw data). The numbers of significant associations for each trait are summarized in Table 3. The majority of traits with associated SNPs were based on genomic groups and MST genotypes, with only continental origin of “Europe” and source of “cow” (excluding milk products) resulting in any additional associations. In the two latter cases, no associations with 100% sensitivity and specificity were observed. Since the MST33 group contained recent outbreak strains, the dataset was analyzed in more detail. No associations with 100% sensitivity and specificity were observed in the MST33 group alone; however, when the closely related MST32 genotype was included, eight such associations could be observed. The majority of SNPs that were specific for the MST33/32 strains were synonymous and did not result in an altered amino acid sequence, with two exceptions: the gene encoding the GIY-YIG catalytic domain protein (group_3567, corresponding to CBU_1112 in RSA493, see Table 2) contained a base substitution that resulted in a premature stop codon at residue 46, and the gene encoding the mannan-binding (MVL family) domain protein described above contained a base substitution resulting in a stop codon at residue 32. In summary, both methods (BPGA subset analysis and Pan-GWAS) add further evidence to the existence of a GG-specific genome content in *C. burnetii*, which is mainly achieved by missense mutations resulting in reductive evolution.

**Table 3:** Summary of Pan-GWAS results

Trait	Total # of associations	# of associations with 100% Sensitivity/Specificity	Comment
Europe	168	0	
Cow tissue	13	0	
GG I	83	3	Same results for MST16
GG II_all	148	0	Includes MST33,32,18,25
GG IIa only	34	4	Includes MST18 and MST25
GG IIb only	152	8	Includes MST33 and MST32
MST18	24	1	
MST33	110	0	
GG III	215	4	Same results for MST20
GG IV	300	8	
GG V	114	44	Same results for MST21
GG VI	123	123	Same results for Rodent source and MST-DG

SNPs that were associated with a particular trait were obtained using the Scoary script on Roary output data. Traits analyzed were Genomic Group, MST genotype, Country of origin, Continent of origin, Host, Human disease type. Only traits with significant associations (Benjamini_Hochberg_p < 10^-3^) are reported

## Discussion

In this study, we provide the first whole genome data for *C. burnetii* obtained in the United Kingdom. We sequenced DNA samples from ruminants after successfully establishing an immunoaffinity method for isolating *Coxiella* from complex samples. Pure culture could not be obtained, mostly due to the presence of contaminating (fast-growing) microorganisms. However, the *C. burnetii* specific DNA content was significantly enriched in DNA samples after immunoaffinity capture (data not shown). All four bovine placenta samples and the sample from the sheep placenta were MST20, which has already been demonstrated to be present in a dairy goat herd in the UK [52]. It is the only MST type currently circulating in bovine milk in the USA [24, 49, 53] after it replaced MST16 genotypes [54], and has so far only been found in cows, sheep, goats, and human tissue in Europe and North America [55]. One goat sample, Cb_D2 was MST32, one of the rarer found genotypes (three entries in the MST database from France, Germany and Austria), and with strain Cb109 as the only sequenced representative to date. WGS data of strain Cb109 contained 257 contigs, whereas the genome for Cb_D2 assembled into 111 contigs (in line with other GG II-b isolates), thereby providing a much improved draft genome. Most interestingly, we found that the epidemic MST33 genotype is present in the UK, with goat placenta samples Q540, Cb_D8, and Cb_D10, the latter two originating from the same farm, representing the first reported cases of this kind. The MST33 was the most commonly found genotype in clinical samples from humans, goats and sheep in the Netherlands in a sampling period that coincided with a drastic increase in the number of Q fever cases between 2007 and 2010, and the outbreak was therefore assumed to be linked to goat farms in close proximity to the human population [23]. A review into goat farming practices in the UK could reveal whether or not a similar outbreak situation as the one observed in the Netherlands could occur.

We also assessed the phylogenetic relationship of the UK isolates and published *C. burnetii* isolates with whole genome data (76 in total at time of submission) using the whole genome alignment Harvest Suite tools. The ParSNP tree grouped isolates according to the *in silico* genotyping results, but provided better resolution by detecting differences between strains that belong to the same MST genotype. The tree determined that isolate Cb171_QLymphoma, which could not be assigned to an MST genotype because only four out of ten MST alleles could be amplified, was related to Cb196_SaudiArabia. The SNP alignment also showed that isolate Cb175_Guyana, a MST17 genotype that clusters with MST16 of GG I in MST trees (see Fig. 2a and Fig. 1 in reference [20]), has a very distinct SNP profile compared to other GG I isolates. We therefore suggest that this isolate should be considered to belong to a separate lineage. This is also supported by the large number of non-synonymous mutations in 397 genes compared to the NM-I reference strain and published phylogenetic trees [56]. Our gene conservation analysis confirmed the loss of the T1SS region in this isolate (data not shown). More sampling in French Guyana and other parts of South America is required to determine the evolutionary history of the MST17 genotype and putative related genotypes, if these can be found.

The whole-genome alignment results also suggested that GG II is divided into two subgroups, which we have termed GG II-a and GG II-b (see Fig. 1). GG II-a is represented by MST18 and MST25 genotypes, whereas GG II-b contains MST33 and MST32 genotypes. The MST tree created in this study (Fig. 2a) confirms the existence of GG II-a, which also includes additional genotypes (MST 22, 23, 29) that have not yet been fully sequenced. Genotypes MST32 and MST24 seem to form a separate cluster from another cluster containing MST33; however, our pan genome analysis suggested that these two clusters have very similar genome content and have therefore collectively been grouped into GG II-b. Variability in the genome content of subgroup GG II-b (see Fig. 4 and Additional file 12: Figure S7) might be achieved through higher rates of genomic rearrangements due to the presence of a higher number of transposable elements (see Kuley *et al.* [48] and references therein for an in-depth discussion of the effects of transposon-mediated recombination), as indicated by a higher number of contigs in the genome assemblies that was also seen in our draft genome assemblies of the UK goat samples (see Table 1).

GG IV can also be divided into subgroups. MST8, which has been found in Europe and the USA, formed one sub-clade in the SNP tree, whereas the remaining isolates in GG IV were all isolated from other continents. A microarray study performed Beare *et al*. [21] suggested that the original classification of GG IV needed revising, and their study assigned strain Q321 to a novel genomic group termed GG VII. Vincent *et al*. proposed further divisions into GG VII to X [57]. The MST tree shown in Fig. 2a suggests that the genetic diversity within GG IV is even higher, with many additional sub-branches being visible that contain MST genotypes without a sequenced representative. Interestingly, the vast majority of isolates that have been deposited in the MST database to date belong to GG IV (see Fig. 2b), and most isolates from continents other than Europe and North America belong to this variable genomic group, which suggests that the true genetic diversity of *C. burnetii* worldwide is underreported due to the lack of genotyping data from other parts of the world. This is supported by a study on Australian isolates, which all showed novel genotypes and formed a unique phylogenetic clade [57]. It is noteworthy that isolate AustraliaQD (and its phase variants) did not cluster with the other Australian isolate AuQ01 in our SNP tree, but was assigned to GG I, which supports the suggestion that the AustraliaQD sample might have been contaminated with the Nine Mile strain DNA before sequencing [57].

We had included *in silico* genotyping analyses as a means to assess the reliability of coreSNP-based phylogenies. Compared to the latter, the genotyping methods were much more sensitive to problems with low sequence quality. Nevertheless, the MST genotype of all previously genotyped isolates was confirmed by our method, except for strain Cb196_SaudiArabia, which has been described as MST51 [58], but which returned MST4 in our analysis. Similarly, strain Dugway 5J108–111 was originally assigned to MST20 [59], but it did not cluster with the other MST20 isolates and returned a novel MST type in our *in silico* analysis and the one performed by Hornstra *et al*. [20], Two other sequenced Dugway isolates (7E65-68 and 7D77-80) showed the same MST genotype as Dugway 5J108-111, confirming that these isolates are not related to MST20. In our hands, acute disease antigen A (*adaA*) genotyping also revealed novel findings: At one point, the *adaA* gene was thought to be associated with *C. burnetii* strains causing acute Q fever [60]. This is now no longer believed to be the case. However, the larger *adaA* region does show variability, which can be used to study microevolution in *C. burnetii* [61]. In this study, two new SNP profiles in the region were identified that were specific for a genomic group. Overall *adaA* typing confirmed the grouping of isolates into genomic groups and was the only genotyping method that was able to distinguish GG II-a and II-b isolates. We also attempted *in silico* MLVA genotyping, which has been shown to be more discriminatory than MST typing [62]. However, we found that our results were not easily comparable with other published MLVA genotypes, an issue that has been highlighted before [63], and therefore MLVA data is not included here. Finally, Olivas *et al*. [49] presented a novel genotyping method to discriminate three distinct sub-genotypes in MST20 isolates. Our results using 82 SNPs confirmed their hypothesis that all European MST20 isolates not typed in the original study belonged to GT_20.1 (see Additional file 8: Figure S4). Three sub-trees within GT_20.1 were visible; one containing only Scandinavian isolates, one containing the only isolates from France, and one containing one of the US isolate (CMSC1). One of the US genomes (isolate CMCA1) did not assemble well in our hands, but its SNP profile is available in the original study, whereas the other two isolates (CMSC1 and ESFL1 from cow’s milk and soil at a cow dairy farm, respectively) could be assembled and were included in our pan genome analysis. The pan-genome based phylogenetic tree obtained using BPGA was unable to distinguish between European and North American MST20 isolates, whereas a core-genome based tree showed clustering of the two non GT_20.1 isolates ESFL1 and Idaho_Goat_195 (see Additional file 10: Figure S6). The ParSNP tree (Fig. 1 and Additional file 5: Figure S1) also clustered strains 18430, 701CbB1 & Cb_B1, as well as strains Cb_B18 & EV_Cb_BK10 and Idaho_Goat_Q195 & ESFL1 together. More isolates are required to confirm this population structure.

Overall, the results obtained by whole genome alignment were corroborated by our pan genome analysis as a measure for gene conservation and pseudogenization. Due to the use of mostly draft genomes, existing polymorphic variants rather than newly acquired gene content showed up as unique and accessory genes in histogram plots. Genomic groups with isolates that clustered tightly in the SNP tree showed the least variable genome content. Similarly, genomic groups with the highest SNP densities, especially GG V, also showed the lowest level of gene sequence conservation. As before, GG IV as a subgroup had the smallest number of core genes. However, splitting GG IV into subgroups as done with GG II and repeating the subset analysis once more genomes become available for each subgroup would most likely result in a bigger core genome content than currently observed for this genomic group. It has to be mentioned that the numbers for the core genome for the species as a whole were dependent on the protein identity setting in the pan genome analysis (see Methods section), and the numbers reported here at a threshold of 90 % are most likely an underestimate; however, this was a measure to reduce false positives in the list of core genes, which can be used to inform the development of new diagnostics and vaccine targets.

As pointed out before, all genes labelled as “new” or “unique” in the pan genome analysis were in fact polymorphic variants due to SNPs, whereas “absent” or “missing” genes were mostly truncated versions due to introduction of stop codons by a SNP or miss-annotated genes with different translational start codons. When analyzing these missing genes, we found some that were unique markers for a genomic group. The Fic-domain (filamentation-induced by c-AMP) protein that is specifically absent in GG I only might be a T4SS substrate, since one of the three Fic domain proteins that are annotated in RSA493 has been confirmed to be secreted via the T4SS and is thought to be involved in posttranslational modification of host molecules [64]. Overall, a significant proportion of GG-specific genes encoded T4SS substrates, such as ankyrin repeat domain-containing proteins (Anks) and other confirmed effectors [8]. This variation in effector repertoire in different strains has been observed before (see Background) and is thought to be the result of ongoing patho-adaptation of *C. burnetii*. This study confirms this finding in the context of genomic groups. The T4SS itself was part of the core genome (data not shown). The majority of the genes that were absent from GG V only were plasmid genes, which is in line with the fact that this genomic group has integrated only a subset of (potentially essential) plasmid genes into their chromosomes [21]. Despite the presence of several predicted membrane proteins in the GG-specific genome content, only one out of 169 identified immunoreactive proteins [65] was found to be specifically absent in one GG. We found that 111 of these proteins were part of the core genome, and another 28 were present in soft core. However, some strongly immunoreactive proteins such as *tuf-2* (CBU_0236) and *groEL* (CBU_1718) were only present in 28 and 24 isolates, respectively, and thus, differences in antigenic profile and the existence of different serotypes of *C. burnetii* cannot be excluded.

Other potential virulence and survival factors are also among the GG-specific genome content: the secreted Cu/Zn superoxide dismutase SodC is truncated in all three Dugway isolates of GG VI, as already described for strain Dugway 5J108-111 [66]. The enzyme plays an important role in intracellular survival and virulence by detoxifying exogenously derived superoxide, and *sodC* mutants of many intracellular bacterial pathogens have been shown to be attenuated [67–70]. In *Coxiella*, the SodC enzyme of the NM-I strain (CBU_1822) has been shown to be enzymatically active and could complement the H_2_O_2_-susceptibility of a *sodC* mutant in *Escherichia coli* [71], but no *C. burnetii* mutant has been characterized to date. This supports a possible attenuation of Dugway isolates due to failure to prevent a lethal oxidative burst by the innate immune response in absence of functional SodC. Full and partial deletions of open reading frames in MST33 (GG II-b) isolates within peptidoglycan genes (CBU_1101-1112) and O-antigen synthesis gene CBU_0691, as well as O-antigen synthesis gene CBU_0686 in MST20 (GG III) isolates has already been described [48], but the effect of these mutations on the expression of these cell wall components has not yet been detailed. A putative mannan-binding protein (MVL) is also absent in MST33 isolates only. However, the encoding *mvl* gene, corresponding to positions 1086949 to 1086509 in the RSA493 reference genome (accession # AE016828), is not annotated as an ORF in the curated genome, but is annotated in other draft genomes. It remains to be elucidated if the MVL proteins are indeed produced by *C. burnetii*.

Finally, pan-genome wide association studies revealed mainly genotype-specific associations. The associations specific to Europe isolates are interesting; however, none of the statistically significant genes produced >90% scores for both sensitivity and specificity. No association with animal source or disease outcome was found, apart from “cow” as source of isolation. However, this group of strains only included one non-MST20 isolate, and therefore, these associations mirror the ones seen in GG III / MST20. It has been noted before that cattle isolates are rarely associated with human disease [72], and a recent study found that two isolates from cattle induce higher pro-inflammatory cytokine release from human peripheral blood mononuclear cells than other animal isolates [73]. The same isolates used in this study were later genome sequenced [48], and both isolates were part of MLVA genotype CbNL12, which we found to correspond to MST20. However, another MST20 isolate from sheep used in the study failed to induce the same proinflammatory response as the MST20 isolates from cattle, which confirms the lack of a genetic basis for these phenotypic differences. The relatively large number of associations observed in GG IV is at odds with the large overall variability in this genomic group, which therefore suggests that this lineage that has been isolated from many different parts of the world might be evolving at a slower rate than other lineages.

## Conclusions

In summary, our data suggest that patho-adaptation and evolution in *C. burnetii* is mainly achieved by point mutations resulting in truncated proteins or proteins with C-terminal polymorphisms. This seems to mostly affect membrane proteins, T4SS effectors such as ankyrin repeat domain-containing proteins, and transporters, thereby adding evidence to the hypothesis that isolates may differ in their antigenic profiles and therefore interact differently with the host immune system [74]. We also found that isolates belonging to the same genomic group were closely related to each other, supporting a model of evolution by clonal expansion where a geotype (genotype specific to a geographical location) has successfully spread to other locations, including rapid inter- and cross-continental spread such as the one observed for GG III (MST20) isolates [49]. Finally, the fact that members of the same genomic group which differ in their date of isolation by many years or even decades (see Additional file 4: Data set S1-A) share a similar SNP profile, and that many of the truncated or polymorphic proteins resulting from these SNPs contain a single frameshift indicates a recent origin and thereby suggests a slow rate of reductive evolution. Overall, our results increase our understanding of the global genetic diversity of this pathogen and provide new insights into the evolution of virulence and other traits, which is essential for the development of new diagnostics, vaccines and therapeutics.

## Methods

### Isolation of *C. burnetii* DNA from tissue

All samples were handled under biosafety level 3 (BSL-3) conditions. Materials included placental tissue from abortions in ruminants in the UK (from two sampling periods processed in two separate batches). Isolation of *C. burnetii* from placenta tissue was performed using an immunoaffinity method in order to enrich *C. burnetii* DNA from the non-sterile environment. A polyclonal goat Anti-*Coxiella* antibody, which was raised against *C. burnetii* isolate LANE (ST12 group) and which is commonly used in the UK to detect both phase I and phase II antigens [75], was coupled to 5 mg of M-270 Epoxy magnetic beads using the Novex Dynabeads® Antibody Coupling Kit (LifeTechnologies) according to the manufacturer’s recommendations. A thumbnail sized piece of placenta was passed through a 40 μm Corning® cell strainer (Sigma Aldrich) into 3 ml RPMI tissue culture medium using a syringe plunger. Next, 500 μl of each placenta homogenate was centrifuged and re-suspended in 1 ml 0.1 % Triton X-100. Samples were incubated at room temperature for 10 mins to lyse cells, subsequently washed with 1 ml PBS and re-centrifuged. Pellets were re-suspended in 1 ml PBS. For immunoaffinity capture, 2 mg of magnetic beads coated with goat anti-*Coxiella* LANE antibody were added to each tube (=200 μl of a 10 mg/ml suspension), and samples were incubated at 37°C with shaking of 200 rpm horizontally for 20 hrs. Next, tubes were placed on a magnetic stand and unbound cells were removed by aspiration. The beads with bound cells were washed three times with PBS and re-suspended in 550 μl of PBS. Bound and unbound fractions were stored in 15 % glycerol at -80°C until further use.

Genomic DNA from bound cells was extracted using the GeneElute Chromosomal DNA extraction kit (Sigma Aldrich), following the protocol for Gram-positive bacteria including an over-night incubation step at 56°C in proteinase K. All DNA samples were ethanol precipitated, sterility tested and re-suspended in 50 μl EB buffer (10 mM Tris-Cl, pH 8.5) before removal out of BSL-3. The DNA quality and concentration was assessed by both Nanodrop and Qubit measurements, and the *Coxiella* DNA content was assessed by standard PCR and Taqman PCR targeting the *com1* gene [76].

### Genome Sequencing and assembly

Sequencing libraries were prepared using a Nextera XT DNA library preparation kit according to the manufacturer’s instructions. Sequencing was performed on an Illumina MiSeq V2 flowcell generating eight million 150-bp paired end reads, with the exception of sample Cb_D1, which was sequenced at 250-bp read length. Illumina adapters were removed and sequences quality trimmed using ea-utils [77]. SPAdes (version 3.7.1) [78] was used to perform a de-novo assembly of the samples.

### Genome annotation and remapping

Fasta sequences of all 76 assembled sequences were annotated using both the web-based RAST (Rapid Annotation using Subsystem Technology) server [79], as well as the command line tool Prokka [80]. The genomes of the nine UK isolates were remapped onto the RSA493 reference sequence using BWA Version: 0.7.12-r1039 [81], and the mean coverage ranged between 69 and 166 times, assessed by using Qualimap [82]. Variants were called using Snippy Version 3.2. [83] using the NM-I RSA493 genome as a reference. Vcf files were merged and the resulting SNPs matrix can be seen in Additional file 4: Data set S1-G.

### Genotyping

All sequenced and publicly available genomes were included in the *in silico* genotyping analyses using the Clone Manager Suite (Sci-Ed Software). Plasmid types were assigned using QpH1 and QpRS specific primers, respectively, as described by Zhang *et al*. [84]. Acute Disease Antigen A (*adaA*) typing, was performed *in silico* using primers L4 nested and R4 nested, as described by Frangoulidis *et al*. [61]. Multispacer sequence typing (MST) of 10 published MST alleles was performed using primers described by Hornstra *et al*. [20] and included spacers Cox2, Cox5, Cox18, Cox20, Cox22, Cox37, Cox51, Cox56, Cox57, and Cox61. Numbers for each allele were assigned using a web-based MST database [85].

### Phylogenetic analysis

The Harvest Suite tools Parsnp and Gingr were used for whole genome alignment, SNP density visualization, and establishing the phylogenetic relationship of strains [86]. SNPs were exported in .vcf format, and the output can be seen in Additional file 4: Data set S1-H. A phylogenetic tree was also constructed using the *in silico* MST alleles. The resulting sequences were concatenated and aligned using the SeaView alignment editor. Previously published MST sequences (MST1-55 at time of submission), were also included in the study. A PhyML tree for known MST alleles was created from variable sites in the SeaView alignment using 100x bootstrap iterations, and trees were analyzed in FigTree graphical viewer. To reconstruct the MST20 phylogeny, nucleotides at 82 sites as defined in Table S2 in Olivas *et al.* [49] were extracted from our whole genome sequences (the SNP matrix can be found in Additional file 4: DataFile S1 _ sheet I) and resulting SNP sequences were uploaded into the SeaView Aligner. A parsimony tree using the inbuilt dnapars algorithm was created using 5x randomized sequence order, bootstrap with 100 replicates, and resulting in 83 steps using 82 sites (30 informative). The RSA493 reference was used to root the tree. All other trees were rooted along the branch leading to GG IV, which corresponds to the position of the root as determined by Pearson et al [87].

### Comparative studies

The genomes of all published and newly sequenced isolates were compared to the NM-I reference strain using the SEED viewer [79] function for a sequence based comparison. The same reference strain genome as used for mapping of sequencing reads had been uploaded in FASTA format and annotated by RAST to allow genuine side-by-side comparisons. Heat maps were created by assigning a scale in shades of grey for the resulting sequence identity data for each gene present in NMI.

Pan-genome analysis was performed using both the pan-genome analysis (BPGA) pipeline [88] and the Perl pipeline Roary [89]. Protein fasta files and gff files created were used as input files, respectively. As before, the output of the de-novo annotation of the RSA493 reference strain genome was included to allow genuine side-by-side comparisons without any annotation bias. Frameshift mutation in the draft genomes had not been fixed and, therefore, affected genes were often annotated as several fragmented proteins, which created bias during the comparative analyses and in this case did not allow the differentiation between “new genes" in the true sense and polymorphic variants due to these frameshifts. Pan-genome analysis in BPGA was performed using USEARCH clustering and 500 permutations at each step of genome addition during pan-genome profile analysis. In order to test and validate the two different pan-genome algorithms (BPGA vs. Roary), we performed a series of analyses on a subset of 41 genomes representing all genomic groups and their subgroups. We used two different gene annotation outputs (RAST vs. PROKKA) as well as three different threshold settings for protein similarities (50%, 90%, and 95%) during clustering. The number of genes assigned to reside within the core genome ranged between 1132 and 1428, and was inversely related to the similarity threshold setting, with fewest core genes found at 95% protein similarity (data not shown). The effect of the similarity threshold setting was more obvious in the BPGA dataset compared to the Roary data, which uses a different clustering algorithm. Plotting the frequency of genes according to blast percentage identity revealed that 87% of protein clusters had identities of >90% (data not shown). Core-Pan-genome plots also confirmed that the changes in the threshold setting affected the BPGA output, but less so the Roary output, and that Prokka and RAST annotation inputs gave similar results (data not shown). In both types of datasets, a large number of genes were assigned to be unique to only one strain. The number of these “unique” genes was lower in the Prokka-annotated datasets, which also had a lower total number of proteins in the input, and was reduced at lower similarity threshold settings, particularly in the BPGA datasets (data not shown).

For analysis of the 67 final genomes, which excluded passage variants and only used the latest sequence data for isolates that have been sequenced twice, we used only Prokka annotations as input due to the perceived more conserved assignment of open reading frames compared to RAST (see # of ORFs in Table 1) and overall comparable outputs. The protein similarity threshold was set to 90% as lower settings increased to occurrence of false positives in the core genome lists. Subset analysis was performed on seven sets of strains representing GG I to GG VI, excluding strain Cb175_Guyana. Phylogenetic analyses in BPGA were performed using default parameters. Gene enrichment analysis was performed by uploading the protein sequences of the core-, accessory-, unique- and exclusively absent genome (BPGA output; Prokka input, 90% similarity threshold) into the KOBAS 3.0 web server [90] for annotation of genes with Uniprot IDs, using *C. burnetii* strain RSA493 as a reference sequence. The extracted Uniprot IDs were then uploaded into the PANTHER classification system server for gene list analysis [91]. The Roary output was used to perform a pan-genome-wide association studies using the Scoary script [92] and various phenotypic traits of the strain collection as query.

## Additional files

Additional file 1: **Table S1.** Coverage data for the nine UK *C. burnetii* genomes sequenced in this study after remapping of sequence reads onto the NM-I reference genome. (PDF 339 kb)

Additional file 2: **Table S2.** Predicted numbers and effects of all genetic variants of the nine UK *C. burnetii* genomes sequenced in this study compared to the NM-I reference genome. (PDF 23 kb)

Additional file 3: **Table S3.** *C. burnetii* isolates and genome data accessions used in this study. (PDF 448 kb)

Additional file 4: **Data File S1.** Raw data for *in silico* MST genotyping and pan-genome analyses. Data Sheets A) Strain Overview; B) *In silico* MST genotyping results; C) Gene Sequence Identity Scores; D) BPGA results _ all strains; E) BPGA results _ Genomic-Group-specific proteins; F) Scoary output _ pan-GWAS significant associations; G) snippy output_merged vcf file; H) ParSNP output _ vcf file; I) MST20_SNP_Matrix. (XLSX 12334 kb)

Additional file 5: **Figure S1.** Phylogenetic relationship of 76 sequenced *C. burnetii* isolates based on core-SNPs. SNP-based phylogenetic relationship (left-hand side) and SNP density plot (right hand side) of all available *C. burnetii* genomes established with Parsnp and visualized with Gingr. SNPs are highlighted in magenta in the density plot, whereas highly conserved regions are highlighted with grey shading. The tree was rooted along the branch leading to GG IV (see Methods). (TIF 6933 kb)

Additional file 6: **Figure S2.** Novel SNP profiles and their position within the *adaA* region. SNP profiles (A) and sequence alignment of the *in silico* generated *adaA* regions (B) were obtained after progressive MAUVE alignment using strain RSA493 (GG I) as a reference. SNP_orig_ = GG II-a, SNP_V2_ = GG II-b, SNP_V3_ = GG III. Note that the original SNP within the *adaA* CDS described by Frangoulidis *et al.* (PLoS ONE 8:e53440, 2013, doi: 10.1371/journal.pone.0053440) corresponds to position 2097 in the *adaA*_Ref._ region. Unique, identifying SNPs are highlighted as colored vertical lines in panel B). (TIF 1293 kb)

Additional file 7: **Figure S3.** Summary of genotyping and phylogenetic analyses. The ParSNP tree obtained after whole-genome alignment (see Fig. 1) was complemented with data from *in silico* plasmid typing, Acute Disease Antigen A (*adaA*) typing, and Multi-Spacer Sequence (MST) typing. Assumed or published data (shown in brackets) was used when typing results were inconclusive or data was missing. (TIF 2679 kb)

Additional file 8: **Figure S4.** Phylogenetic relationship between MST20 (GG III) isolates. A maximum-parsimony tree was reconstructed based on 82 SNPs defined by Olivas *et al.* (Microb. Genom. 2016, 2(8):e000068) using RSA493 as a reference and to root the tree. MST20 sub-genotypes (GT_20.1-3) as defined by Olivas *et al*. are colour coded. (TIF 301 kb)

Additional file 9: **Figure S5.** Core-Pan-genome plots and gene frequency plots for 67 *C. burnetii* genomes. Proteins annotated using PROKKA were used as input files for BPGA (A&C) and Roary (B&D) pan-genome analyses. The protein similarity threshold for protein clustering was 90% in all cases. Core-Pan-genome plots (A&B) and gene frequency plots (C&D) are shown. (TIF 368 kb)

Additional file 10: **Figure S6.** Phylogenetic relationship of 67 *C. burnetii* isolates based on partial genome content. A) Core genome-based tree determined by Roary, B) Core genome-based tree determined by BPGA, C) Accessory (binary) genome-based tree determined by Roary, and D) Pan genome-based tree determined by BPGA. Newick outputs of both BPGA and Roary pipelines were used to draw trees using FigTree, and Genomic Groups were color coded. All trees were rooted along the branch leading to GG IV (see Methods). (TIF 3891 kb)

Additional file 11: **Table S4.** Results of a PANTHER gene enrichment analysis of core and accessory genome contents of 67 *C. burnetii* isolates. Note that no significant enrichment was found in the unique genome. (PDF 29 kb)

Additional file 12: **Figure S7.** New gene plots after BPGA pan-genome subset analysis using groups of isolates according to their genomic group associations. Proteins annotated using PROKKA were used as input files. The protein similarity threshold for protein clustering was 90%. Genomic groups were assigned as seen in Fig. 1. Note that “new” genes represent polymorphic variants due to the presence of SNPs in existing genes rather than newly acquired genes. (TIF 404 kb)

## Data Availability

Whole genome sequences were deposited in NCBI under BioProjects PRJNA430350 and PRJNA506366, as well as in the Sequence Read Archive as studies SRP130048 and SRP170036. Individual GenBank accession numbers for the WGS data are as follows: Q532 = PPFQ00000000.1 ; Q540 = PPFP00000000.1 ; Q545 = PPFO00000000.1 ; Q556 = PPFN00000000.1 ; Q559 = PPFM00000000.1 ; Cb_D1 = RQJU00000000.1; Cb_D2 = RQJT00000000.1 ; Cb_D8 = RQJS00000000.1 ; and Cb_D10 = RQJR00000000.1 .The authors declare that all other data supporting the findings of this study are available within the article and its supplementary information files.

## Abbreviations

*NM-I*: Nine-Mile phase I reference strain (RSA493)
*GG*: Genomic Group
*SNP*: Single nucleotide polymorphism
*MST*: Multispacer Sequence Typing (MST)
*MLVA*: Multiple-Locus Variable number tandem repeat Analysis
*GWAS*: Genome-wide association study
*BPGA*: Bacterial Pan Genome Analysis
*LPS*: Lipopolysaccharide
*T4SS*: Type IV secretion system
