# Supplementary material for: Extensive genome analysis of Coxiella burnetii reveals limited evolution within genomic groups
Source: BMC Genomics. 2019 Jun 5;20:441. doi: 10.1186/s12864-019-5833-8 (PMC6549354; doi:10.1186/s12864-019-5833-8)

Table S1: Coverage data from the nine UK *C. burnetii* isolates sequenced in this study after remapping of sequence reads onto the NM-I reference genome. Remapping and coverage analysis was performed as described in the Methods section. Deletion events in MST33 isolates (right panel) that have previously been described for related Dutch outbreak strains by Kuley *et al.* (Front Microbiol 8, 2017. doi: 10.3389/fmicb.2017.01526) are highlighted in bold.

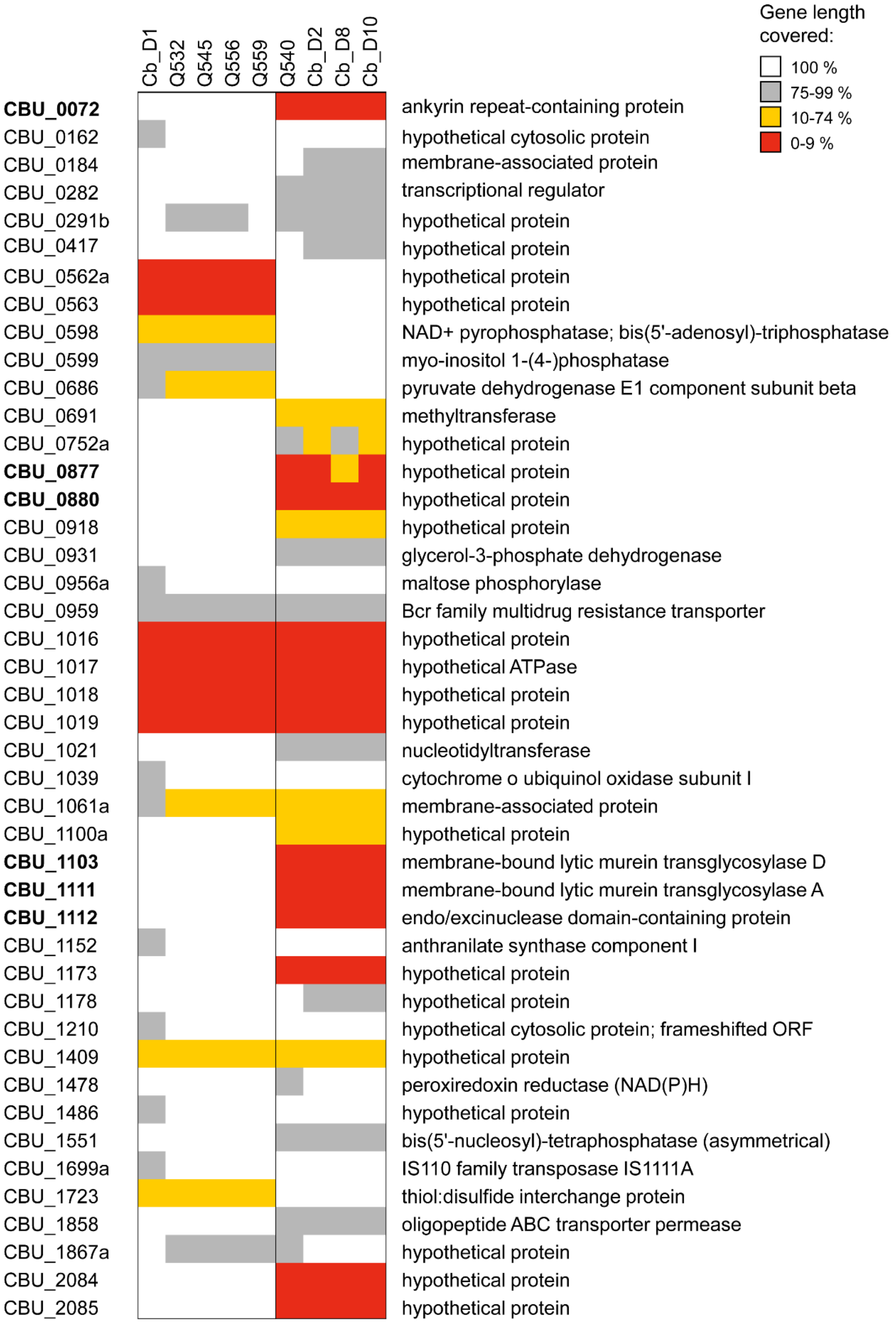

Supplement: Supplementary file 1 — Table S1. Coverage data for the nine UK C. burnetii genomes sequenced in this study after remapping of sequence reads onto the NM-I reference genome. (PDF 339 kb) [file 12864_2019_5833_MOESM1_ESM.pdf]
