# Supplementary material for: Extensive genome analysis of Coxiella burnetii reveals limited evolution within genomic groups
Source: BMC Genomics. 2019 Jun 5;20:441. doi: 10.1186/s12864-019-5833-8 (PMC6549354; doi:10.1186/s12864-019-5833-8)
Supplement: Supplementary file 2 — Table S2. Predicted numbers and effects of all genetic variants of the nine UK C. burnetii genomes sequenced in this study compared to the NM-I reference genome. (PDF 23 kb) [file 12864_2019_5833_MOESM2_ESM.pdf]

Table S2: Predicted numbers and effects of all genetic variants of the nine UK *C. burnetii* isolates sequenced in this study compared to the NM-I reference genome. SNPs were called in relation to the Nine Mile reference strain using snippy (see Methods).

	GG III					GG II			
	Cb_D1	Q532	Q545	Q556	Q559	Q540	Cb_D2	Cb_D8	Cb_D10
Variant Type									
COMPLEX	47	38	36	36	38	61	57	61	61
DEL	162	155	153	147	152	229	226	227	223
INS	108	93	96	91	92	100	110	106	100
MNP	8	4	7	2	4	7	10	6	2
SNP	1788	1744	1755	1750	1752	2386	2376	2387	2376
Variant Effect									
HIGH	109	101	99	97	100	150	151	149	141
MODERATE	788	750	756	753	758	1016	1012	1013	1007
LOW	428	424	428	423	423	619	619	619	620
Variant Location									
within-CDS	1325	1275	1283	1273	1281	1785	1782	1781	1768
Non-CDS	787	759	764	753	757	998	997	1006	994
**ALL VARIANTS**	**2113**	**2034**	**2047**	**2026**	**2038**	**2783**	**2779**	**2787**	**2762**

Abbreviations: SNP = Single Nucleotide Polymorphism; MNP = Multiple Nuclotide Polymorphism; INS = Insertion; DEL = Deletion; COMPLEX = Combination of snp/mnp; CDS = Coding Sequence
