# Supplementary material for: Extensive genome analysis of Coxiella burnetii reveals limited evolution within genomic groups
Source: BMC Genomics. 2019 Jun 5;20:441. doi: 10.1186/s12864-019-5833-8 (PMC6549354; doi:10.1186/s12864-019-5833-8)
Supplement: Supplementary file 3 — Table S3. C. burnetii isolates and genome data accessions used in this study. (PDF 448 kb) [file 12864_2019_5833_MOESM3_ESM.pdf]

Table S3: *C. burnetii* isolates and genome data accessions used in this study.

Strain (Synonym)	Source	Country	GG	Genome Release Date	Accession # / Source file	Reference (Sequence)
**RSA493 (NineMile; NM-I)**	Tick	USA	I	2003	**chr:** AE016828.2 **pl:** AE016829.1	[1]
**MSU Goat Q177 (Priscilla)**	Goat cotyledon	USA	IV	2006	**pl:** NC_010258.1 /CP000914.1 **WGS:** AAUP02	[2]
**Dugway 5J108-111**	Rodent	USA	VI	2007	**chr:** CP000733.1 **pl:** CP000735.1	[3]
**RSA331 (Henzerling)**	Acute Q fever patient (blood)	Italy	II (a)	2007	**chr:** CP000890.1 **pl:** CP000889.1	[2]
**Q321**	Cow's Milk	Russia	IV	2007	**WGS:** AAYJ01	[2]
**CbuG_Q212**	Human Heart Valve	Canada	V	2008	**chr:** NC_011527.1 /CP001019.1	[4]
**CbuK_Q154**	Human Heart Valve	USA	IV	2008	**chr:** NC_011528.1 **pl:** NC_011526.1	[4]
**Cb109**	Human Heart Valve (doxy^{R})	Germany	II (b)	2012	**WGS:** AKYP01	[5]
**Cb185**	Human Placenta	France	II (a)	2013	**WGS:** CBTH01 (plasmid n.a.)	[6]
**Cb175_Guyana**	Human Heart Valve	French Guyana	(I)	2013	**WGS:** CAVF01	[7]
**Z3055**	Sheep placenta	Germany	II (b)	2014	**chr:** NZ_LK937696.1 /LK937696.1(plasmid n.a.)	[8]
**Namibia**	Goat abortion	Namibia	IV	2014	**chr:** NZ_CP007555.1 **pl:** NZ_CP007556.1	[9]
**Cb_C2**	Goat milk	France	I	2014	**WGS:** CCAJ01	[10]
**Cb_B1**	Cow placenta	France	III	2014	**WGS:** CCAH01	[10]
**EV-Cb_BK10**	Cow vaginal swab	Sweden	III	2014	**WGS:** CCAL01	[10]
**Cb_O184**	Sheep placenta	France	IV	2014	**WGS:** CCAK01	[10]
**EV-Cb_C13**	Goat abortion	Germany	II (b)	2014	**WGS:** CCAM01	[10]
**Cb_B18**	Cow placenta	Denmark	III	2014	**WGS:** CCAI01	[10]
**AuQ01 (Arandale)**	Acute Q fever patient (blood)	Australia	IV	2014	**pl:** NZ_JPVV01000067.1 **WGS:** JPVV01	[11]
**Cb196_SaudiArabia**	Human Heart Valve	Saudi Arabia	IV	2014	**WGS:** CCXO01 (plasmid n.a.)	[12]
**Cb171_QLYMPHOMA**	Human Lymphoma	n.a.	IV	2014	**WGS**: CDBG01 (plasmid n.a.)	n.a.
**Dog_Utad (RSA493_DogUtad)**	Dog uterus (2 isolates)	Canada	V	2014	**WGS:** CCNR01 and CCYB01	[13]
**Ohio**	Milk	USA	I	2014	**SRA:** SRR833203	[14]
**S4**	Sheep	Sweden	I	2014	**SRA:** SRR833204	[14]
**C2**	Hay	Sweden	I	2014	**SRA:** SRR833199	[14]
**Innsbruck (Z2534)**	Goat	Austria	II (a)	2014	**SRA:** SRR833200	[14]
**2338**	Cow	Germany	II (a)	2014	**SRA:** SRR833201	[14]
**Z349-36/94**	Sheep	Germany	II (a)	2014	**SRA:** SRR833206	[14]

Strain (Synonym)	Source	Country	GG	Genome Release Date	Accession # / Source file	Reference (Sequence)
**Scurry (S; Q217)**	Human	USA	V	2014	**SRA:** SRR833205	[14]
**Australia QD (RSA425)**	Human blood	Australia	I	2014	**SRA:** SRR405074	[14]
**M44 (Grita; RSA459)**	Human blood	Italy	II (a)	2014	**SRA:** SRR407385	[14]
**McMaster (Q172)**	Human placenta	n.a.	V	2014	**SRA:** SRR405075	[14]
**NL-Limburg**	Human Aortic Aneurism	Netherlands	II (b)	2015	**WGS:** JZWL01	[15]
**NL3262**	Goat placenta	Netherlands	II (b)	2016	**chr:** NZ_CP013667.1 **pl:** NZ_CP013668.1	[16]
**NLhu3345937**	Human Heart Valve	Netherlands	II (b)	2016	**chr:** NZ_CP014354.1 **pl:** NZ_CP014355.1	[16]
**RSA439_NMII**	Passage variant of RSA493	USA	I	2017	**chr:** CP020616 **pl:** CP020617	[17]
**Turkey_RSA315**	Acute Q fever patient (blood)	Turkey	I	2017	**WGS:** NOLO00000000	[18]
**Dyer_RSA345**	Acute Q fever patient (blood)	USA	I	2017	**WGS:** NOLQ00000000	[18]
**Ko_Q229**	Human Heart Valve	Canada	V	2017	**WGS:** NOLP00000000	[18]
**Dugway 7E65-68**	Rodent	USA	VI	2017	**WGS:** NOLM00000000	[19]
**Dugway 7D77-80**	Rodent	USA	VI	2017	**WGS:** NOLN00000000	[19]
**Idaho_Goat_Q195**	Goat placenta	USA	III	2017	**WGS:** NOLR00000000	[20]
**Ohio314_RSA338**	Passage variant of RSA270	USA	I	2017	**WGS:** NOLS00000000	[20]
**Ohio314_RSA270**	Cow's Milk	USA	I	2017	**WGS:** NOLT00000000	[20]
**California33_RSA329**	Cow's Milk	USA	I	2017	**WGS:** NOLV00000000	[20]
**California16_RSA350**	Cow's Milk	USA	I	2017	**WGS:** NOLU00000000	[20]
**CbCVIC1**	Goat	Netherlands	II (b)	2017	**chr:** CP014549 **pl:** CP014550	[21]
**602 (14160-002)**	Goat	Netherlands	II (b)	2017	**chr:** CP014836 **pl:** CP014837	[21]
**42785537**	Human chronic Q fever	Netherlands	II (b)	2017	**chr:** CP014548 **pl:** CP014547	[21]
**601 (14160-001)**	Goat	Netherlands	III	2017	**chr:** CP014551 **pl:** CP014552	[21]
**18430**	Sheep	Netherlands	III	2017	**chr:** CP014557 **pl:** CP014558	[21]
**701CbB1**	Cow	France	III	2017	**chr:** CP014553 **pl:** CP014554	[21]
**2574**	Cow	Netherlands	III	2017	**chr:** CP014555 **pl:** CP014556	[21]
**Henzerling_Kuley**	Human acute Q fever	Italy	II (a)	2017	**chr:** CP014559 **pl:** CP014560	[21]
**Heizberg**	Human acute Q fever	Greece	II (a)	2017	**chr:** CP014561 **pl:** CP014562	[21]
**Schperling**	Human acute Q fever	Kyrgyzstan	IV	2017	**chr:** CP014563 **pl:** KY271744	[21]
**Scurry_Kuley**	Human liver biopsy	USA	V	2017	**chr:** CP014565	[21]

Strain (Synonym)	Source	Country	GG	Genome Release Date	Accession # / Source file	Reference (Sequence)
**Leningrad-2**	Human Heart Valve	Russia	IV	2017	**WGS:** PDLP00000000.1	[22}
**V525_Tunisia**	Human Heart valve	Tunisia	IV	2017	**SRA:** ERR2303613	[22]
**CMSC1**	Cow's milk	USA	III	2018	**SRA:** SRR3347458	[23]
**ESFL1**	Soil (dairy farm)	USA	III	2018	**SRA:** SRR3347474	[23]
**RSA363**	Passage variant of RSA493	USA	I	2018	**WGS:** NOVH00000000.1	[24]
**RSA514_NMC (NM-Crazy)**	Chronically infected guinea pig	USA	I	2018	**WGS:** NOVG00000000.1	[24]
**RSA425_Australia**	Passage variant of AustraliaQD	USA?	I	2018	**WGS:** NOVJ00000000.1	[24]
**RSA297_Australia**	Passage variant of AustraliaQD	USA?	I	2018	**WGS:** NOVK00000000.1	[24]
**California16_RSA350_Clone2**	Clone 2 of California 16 _RSA350	USA	I	2018	**WGS:** NOVF00000000.1	[24]
**M44_RSA461_Clone1**	Human (blood)	Italy	II (a)	2018	**WGS:** NOVI00000000.1	[24]
**Q532**	Cow placenta	UK	III	2018	**WGS:** PPFQ00000000.1	This study
**Q540**	Goat placenta	UK	II (b)	2018	**WGS:** PPFP00000000.1	This study
**Q545**	Cow placenta	UK	III	2018	**WGS:** PPFO00000000.1	This study
**Q556**	Cow placenta	UK	III	2018	**WGS:** PPFN00000000.1	This study
**Q559**	Sheep placenta	UK	III	2018	**WGS:** PPFM00000000.1	This study
**Cb_D1 (DSTL_1R)**	Cow placenta	UK	III	2018	**WGS:** RQJU00000000.1	This study
**Cb_D2 (DSTL_2)**	Goat placenta	UK	II (b)	2018	**WGS:** RQJT00000000.1	This study
**Cb_D8 (DSTL_8)**	Goat placenta	UK	II (b)	2018	**WGS:** RQJS00000000.1	This study
**Cb_D10 (DSTL_10)**	Goat placenta	UK	II (b)	2018	**WGS:** RQJR00000000.1	This study

Abbreviations: chr = chromosome; pl = plasmid; WGS = whole-genome-sequencing contigs only; SRA = sequence read archive; IP = integrated plasmid; n.a. = not available

REFERENCES:

1. Seshadri R, Paulsen IT, Eisen JA, Read TD, Nelson KE, Nelson WC, Ward NL, Tettelin H, Davidsen TM, Beanan MJ *et al*: **Complete genome sequence of the Q-fever pathogen *Coxiella burnetii***. *Proc Nat Acad Sci USA* 2003, **100**(9):5455-5460.
2. Beare PA, Samuel JE, Howe D, Virtaneva K, Porcella SF, Heinzen RA: **Genetic Diversity of the Q Fever Agent, *Coxiella burnetii*, Assessed by Microarray-Based Whole-Genome Comparisons**. *J Bacteriol* 2006, **188**(7):2309-2324.
3. Beare PA, Unsworth N, Andoh M, Voth DE, Omsland A, Gilk SD, Williams KP, Sobral BW, Kupko JJ, Porcella SF *et al*: **Comparative Genomics Reveal Extensive Transposon-Mediated Genomic Plasticity and Diversity among Potential Effector Proteins within the Genus *Coxiella***. *Infect Immun* 2009, **77**(2):642-656.
4. Beare PA, Porcella SF, Seshadri R, Samuel JE, Heinzen RA: **Preliminary Assessment of Genome Differences between the Reference Nine Mile Isolate and Two Human Endocarditis Isolates of *Coxiella burnetii***. *Ann N Y Acad Sci* 2005, **1063**(1):64-67.
5. Rouli L, Rolain J-M, El Filali A, Robert C, Raoult D: **Genome Sequence of *Coxiella burnetii* 109, a Doxycycline-Resistant Clinical Isolate**. *J Bacteriol* 2012, **194**(24):6939.
6. Million M, Roblot F, Carles D, D'Amato F, Protopopescu C, Carrieri MP, Raoult D: **Reevaluation of the Risk of Fetal Death and Malformation After Q Fever**. *Clin Infect Dis* 2014, **59**(2):256-260.

7. D'Amato F, Eldin C, Georgiades K, Edouard S, Delerce J, Labas N, Raoult D: **Loss of TSS1 in hypervirulent *Coxiella burnetii* 175, the causative agent of Q fever in French Guiana**. *Comp Immunol Microbiol Infect Dis* 2015, **41**:35-41.
8. D'Amato F, Rouli L, Edouard S, Tyczka J, Million M, Robert C, Nguyen TT, Raoult D: **The genome of *Coxiella burnetii* Z3055, a clone linked to the Netherlands Q fever outbreaks, provides evidence for the role of drift in the emergence of epidemic clones**. *Comp Immunol Microbiol Infect Dis* 2014, **37**(5–6):281-288.
9. Walter MC, Öhrman C, Myrtennäs K, Sjödin A, Byström M, Larsson P, Macellaro A, Forsman M, Frangoulidis D: **Genome sequence of *Coxiella burnetii* strain Namibia**. *Standards in Genomic Sciences* 2014, **9**(1):1-9.
10. Sidi-Boumedine K, Ellis RJ, Adam G, Prigent M, Angen Ø, Aspán A, Thiéry R, Rousset E: **Draft Genome Sequences of Six Ruminant *Coxiella burnetii* Isolates of European Origin**. *Genome Announc* 2014, **2**(3):e00285-00214.
11. Walter MC, Vincent GA, Stenos J, Graves S, Frangoulidis D: **Genome Sequence of *Coxiella burnetii* Strain AuQ01 (Arandale) from an Australian Patient with Acute Q Fever**. *Genome Announcements* 2014, **2**(5).
12. D'Amato F, Robert C, Azhar EI, Fournier P-E, Raoult D: **Draft Genome Sequence of *Coxiella burnetii* Strain Cb196, an Agent of Endocarditis in Saudi Arabia**. *Genome Announcements* 2014, **2**(6).
13. D'Amato F, Million M, Edouard S, Delerce J, Robert C, Marrie T, Raoult D: **Draft genome sequence of *Coxiella burnetii* Dog Utad, a strain isolated from a dog-related outbreak of Q fever**. *New Microbes and New Infections* 2014, **2**(4):136-137.
14. Karlsson E, Macellaro A, Byström M, Forsman M, Frangoulidis D, Janse I, Larsson P, Lindgren P, Öhrman C, van Rotterdam B *et al*: **Eight New Genomes and Synthetic Controls Increase the Accessibility of Rapid Melt-MAMA SNP Typing of *Coxiella burnetii***. *PLoS ONE* 2014, **9**(1):e85417.
15. Hammerl JA, Mertens K, Sprague LD, Hackert VH, Buijs J, Hoebe CJ, Henning K, Neubauer H, Al Dahouk S: **First Draft Genome Sequence of a Human *Coxiella burnetii* Isolate, Originating from the Largest Q Fever Outbreak Ever Reported, the Netherlands, 2007 to 2010**. *Genome Announcements* 2015, **3**(3).
16. Kuley R, Smith HE, Janse I, Harders FL, Baas F, Schijlen E, Nabuurs-Franssen MH, Smits MA, Roest HIJ, Bossers A: **First Complete Genome Sequence of the Dutch Veterinary *Coxiella burnetii* Strain NL3262, Originating from the Largest Global Q Fever Outbreak, and Draft Genome Sequence of Its Epidemiologically Linked Chronic Human Isolate NLhu3345937**. *Genome Announc* 2016, **4**(2).
17. Millar JA, Beare PA, Moses AS, Martens CA, Heinzen RA, Raghavan R: **Whole-Genome Sequence of *Coxiella burnetii* Nine Mile RSA439 (Phase II, Clone 4), a Laboratory Workhorse Strain**. *Genome Announc* 2017, **5**(23).
18. Beare PA, Jeffrey BM, Martens CA, Heinzen RA: **Draft Genome Sequences of Three *Coxiella burnetii* Strains Isolated from Q Fever Patients**. *Genome Announc* 2017, **5**(38).
19. Beare PA, Jeffrey BM, Martens CA, Heinzen RA: **Draft Genome Sequences of the Avirulent *Coxiella burnetii* Dugway 7D77-80 and Dugway 7E65-68 Strains Isolated from Rodents in Dugway, Utah**. *Genome Announc* 2017, **5**(39).
20. Beare PA, Jeffrey BM, Martens CA, Pearson T, Heinzen RA: **Draft Genome Sequences of Historical Strains of *Coxiella burnetii* Isolated from Cow's Milk and a Goat Placenta**. *Genome Announc* 2017, **5**(39).
21. Kuley R, Kuijt E, Smits MA, Roest HIJ, Smith HE, Bossers A: **Genome Plasticity and Polymorphisms in Critical Genes Correlate with Increased Virulence of Dutch Outbreak-Related *Coxiella burnetii* Strains**. *Front Microbiol* 2017, **8**(1526).
22. Delaloye J, Pillonel T, Smaoui M, Znazen A, Abid L, Greub G: **Culture-independent genome sequencing of *Coxiella burnetii* from a native heart valve of a Tunisian patient with severe infective endocarditis**. *New Microbes New Infect* 2017.
23. Olivas S, Hornstra H, Priestley RA, Kaufman E, Hepp C, Sonderegger DL, Handady K, Massung RF, Keim P, Kersh GJ *et al*: **Massive dispersal of *Coxiella burnetii* among cattle across the United States**. *Microb Genom* 2016, **2**(8):e000068.
24. Beare PA, Jeffrey BM, Long CM, Martens CM, Heinzen RA: **Genetic mechanisms of *Coxiella burnetii* lipopolysaccharide phase variation**. *PLOS Pathogens* 2018, **14**(3):e1006922.
