# Supplementary figures and images for: Extensive genome analysis of Coxiella burnetii reveals limited evolution within genomic groups
Source: BMC Genomics. 2019 Jun 5;20:441. doi: 10.1186/s12864-019-5833-8 (PMC6549354; doi:10.1186/s12864-019-5833-8)

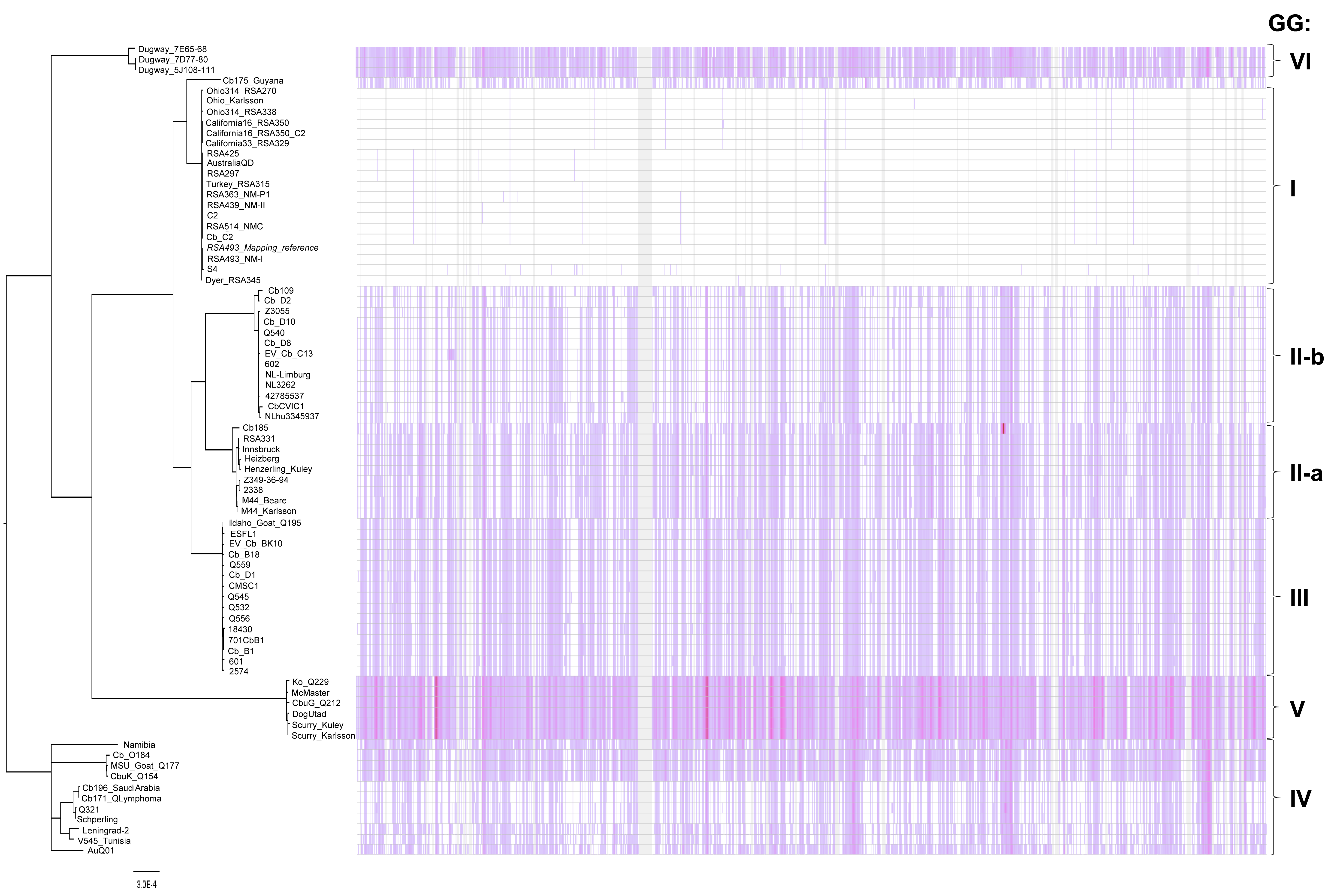

Supplement: Supplementary file 5 — Figure S1. Phylogenetic relationship of 76 sequenced C. burnetii isolates based on core-SNPs. SNP-based phylogenetic relationship (left-hand side) and SNP density plot (right hand side) of all available C. burnetii genomes established with Parsnp and visualized with Gingr. SNPs are highlighted in magenta in the density plot, whereas highly conserved regions are highlighted with grey shading. The tree was rooted along the branch leading to GG IV (see Methods). (TIF 6933 kb) [file 12864_2019_5833_MOESM5_ESM.tif]

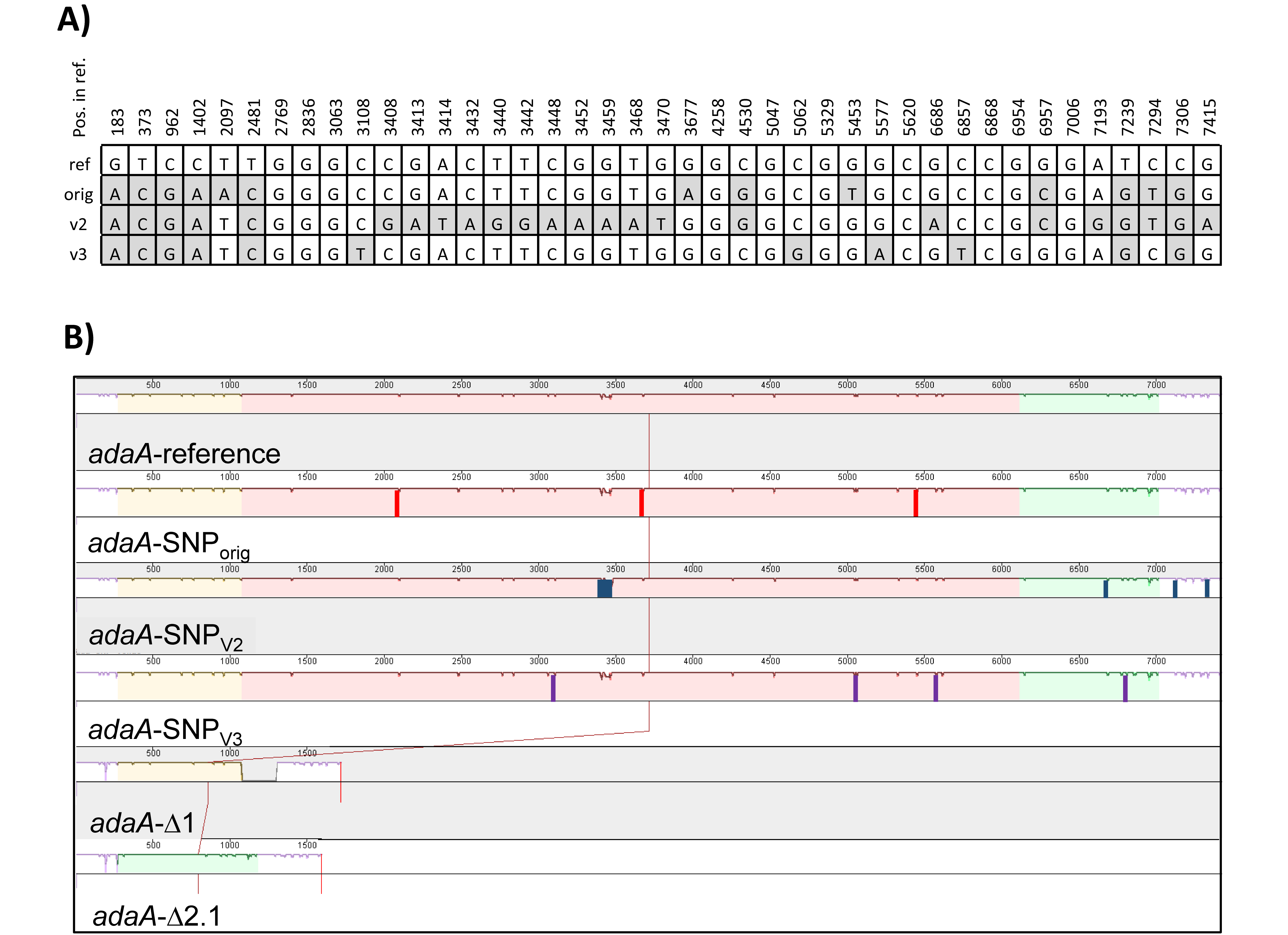

Supplement: Supplementary file 6 — Figure S2. Novel SNP profiles and their position within the adaA region. SNP profiles (A) and sequence alignment of the in silico generated adaA regions (B) were obtained after progressive MAUVE alignment using strain RSA493 (GG I) as a reference. SNPorig = GG II-a, SNPV2 = GG II-b, SNPV3 = GG III. Note that the original SNP within the adaA CDS described by Frangoulidis et al. (PLoS ONE 8:e53440, 2013, doi: 10.1371/journal.pone.0053440) corresponds to position 2097 in the adaARef. region. Unique, identifying SNPs are highlighted as colored vertical lines in panel B). (TIF 1293 kb) [file 12864_2019_5833_MOESM6_ESM.tif]

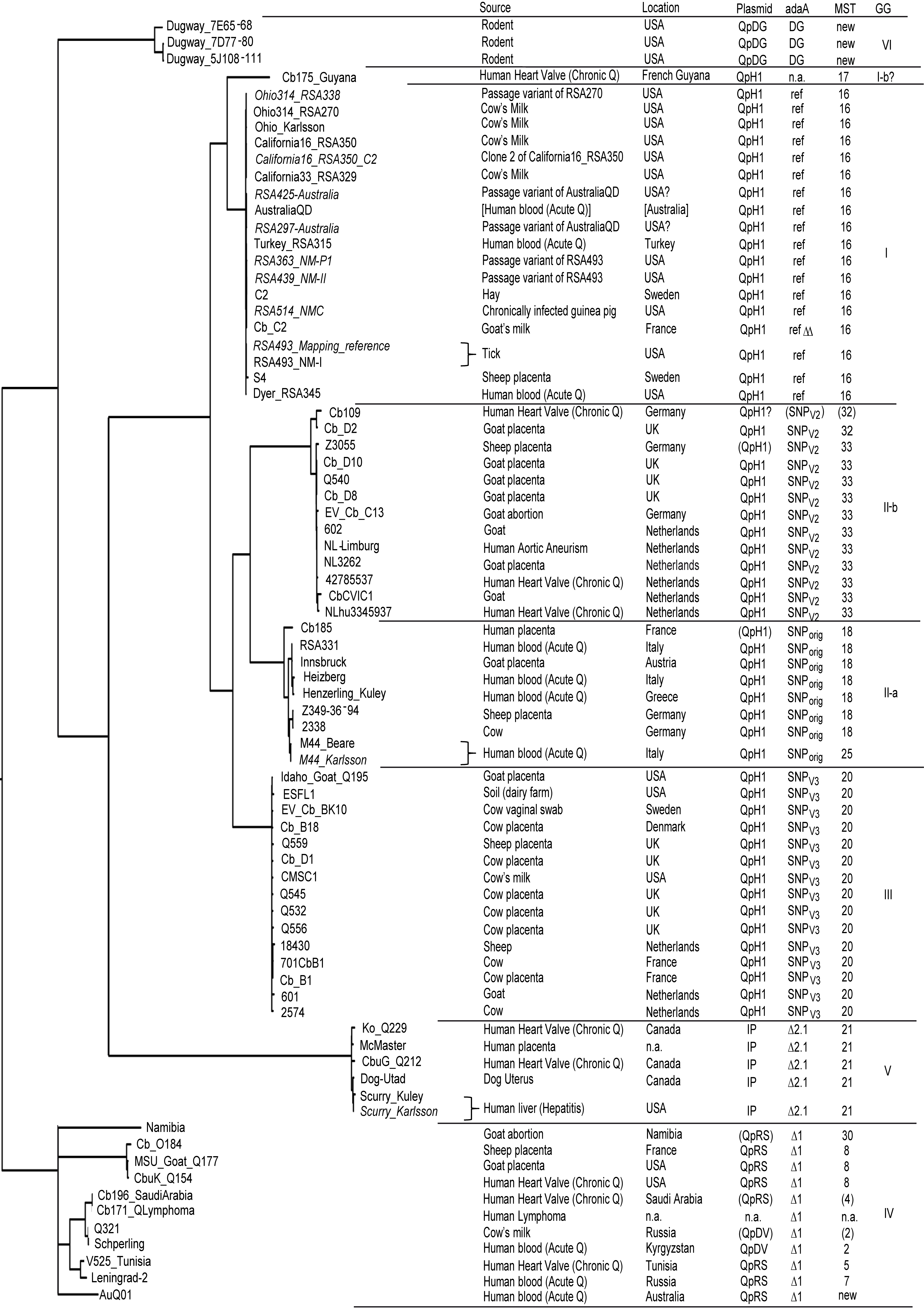

Supplement: Supplementary file 7 — Figure S3. Summary of genotyping and phylogenetic analyses. The ParSNP tree obtained after whole-genome alignment (see Fig. 1) was complemented with data from in silico plasmid typing, Acute Disease Antigen A (adaA) typing, and Multi-Spacer Sequence (MST) typing. Assumed or published data (shown in brackets) was used when typing results were inconclusive or data was missing. (TIF 2679 kb) [file 12864_2019_5833_MOESM7_ESM.tif]

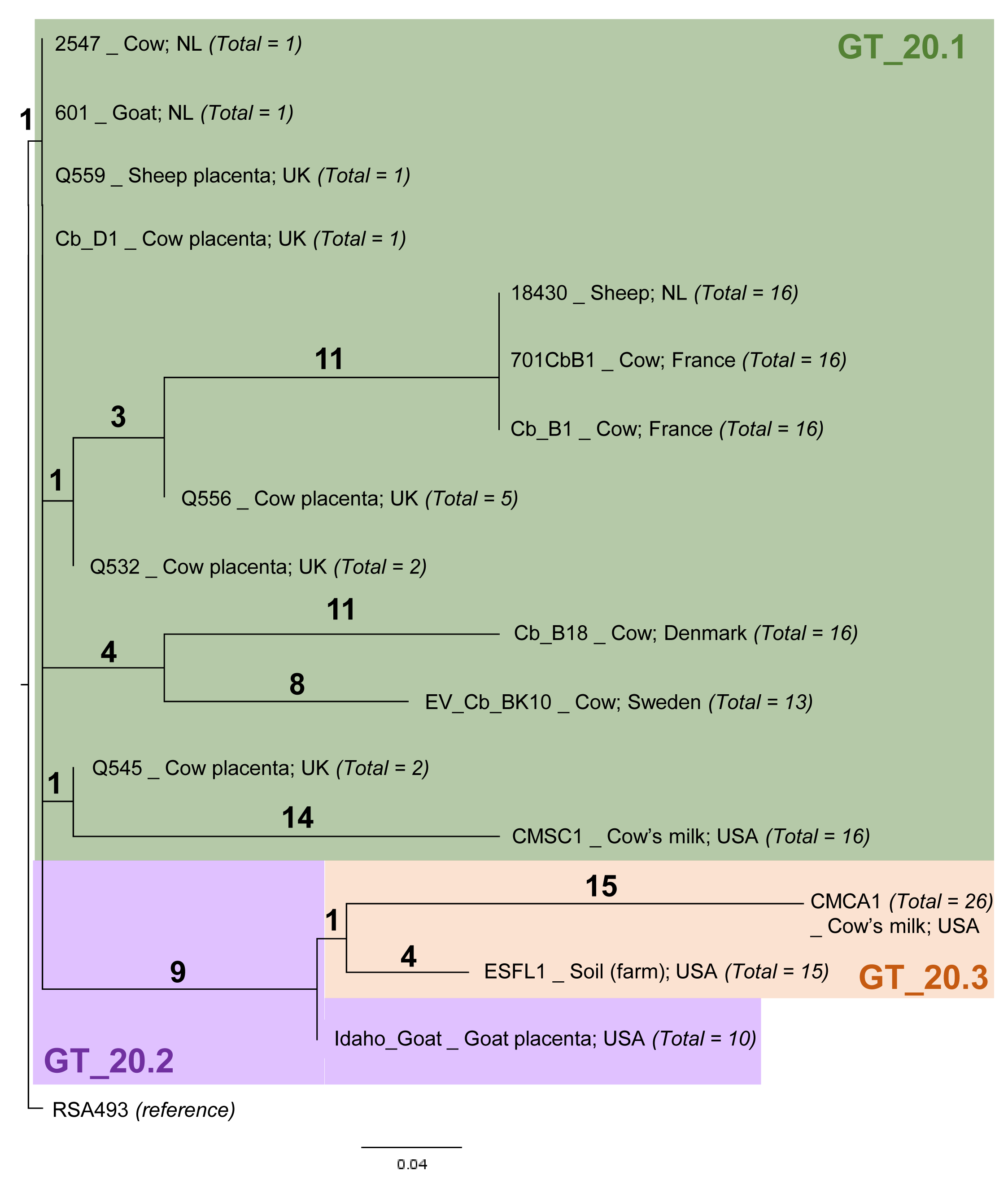

Supplement: Supplementary file 8 — Figure S4. Phylogenetic relationship between MST20 (GG III) isolates. A maximum-parsimony tree was reconstructed based on 82 SNPs defined by Olivas et al. (Microb. Genom. 2016, 2(8):e000068) using RSA493 as a reference and to root the tree. MST20 sub-genotypes (GT_20.1-3) as defined by Olivas et al. are colour coded. (TIF 301 kb) [file 12864_2019_5833_MOESM8_ESM.tif]

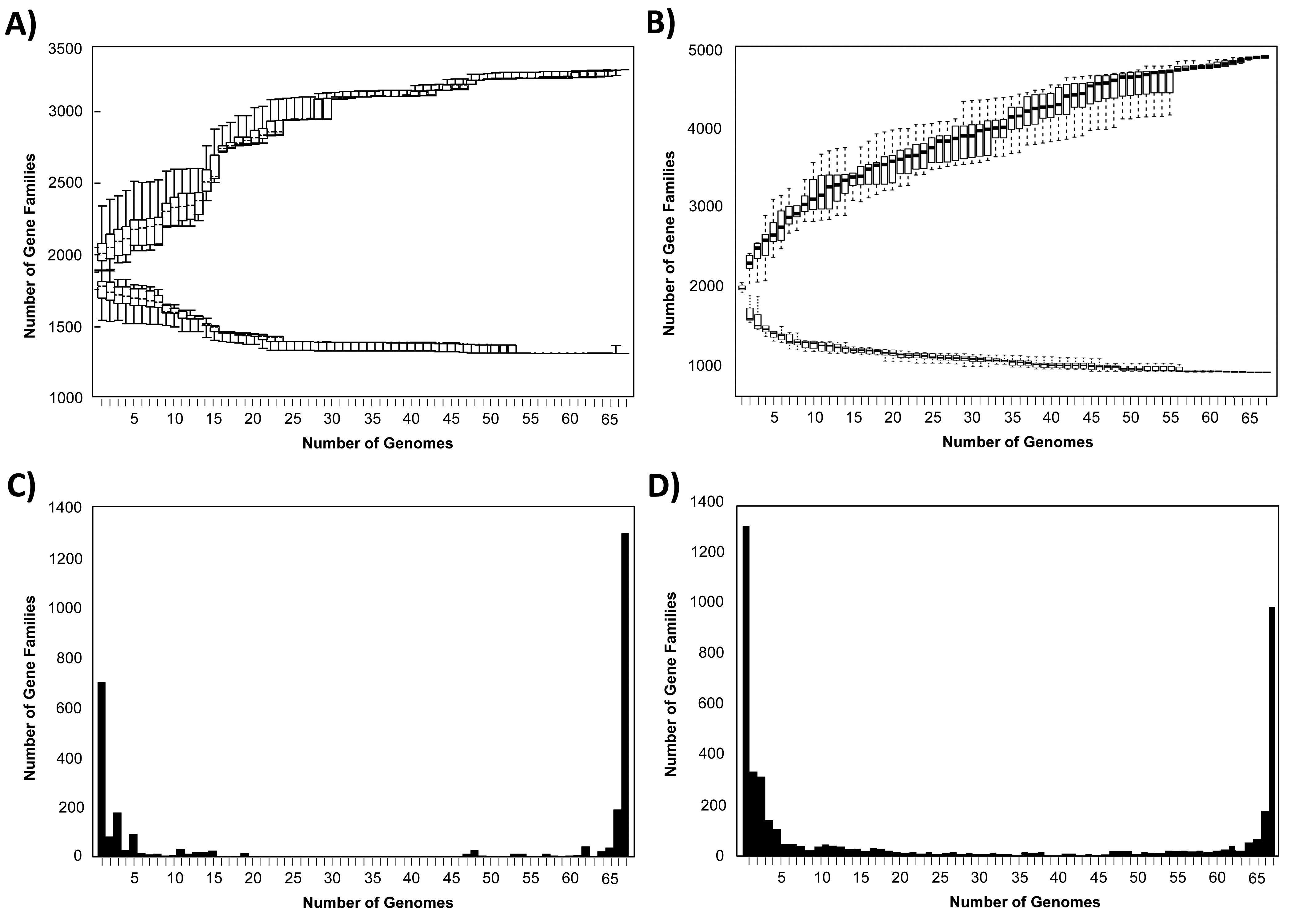

Supplement: Supplementary file 9 — Figure S5. Core-Pan-genome plots and gene frequency plots for 67 C. burnetii genomes. Proteins annotated using PROKKA were used as input files for BPGA (A&C) and Roary (B&D) pan-genome analyses. The protein similarity threshold for protein clustering was 90% in all cases. Core-Pan-genome plots (A&B) and gene frequency plots (C&D) are shown. (TIF 368 kb) [file 12864_2019_5833_MOESM9_ESM.tif]

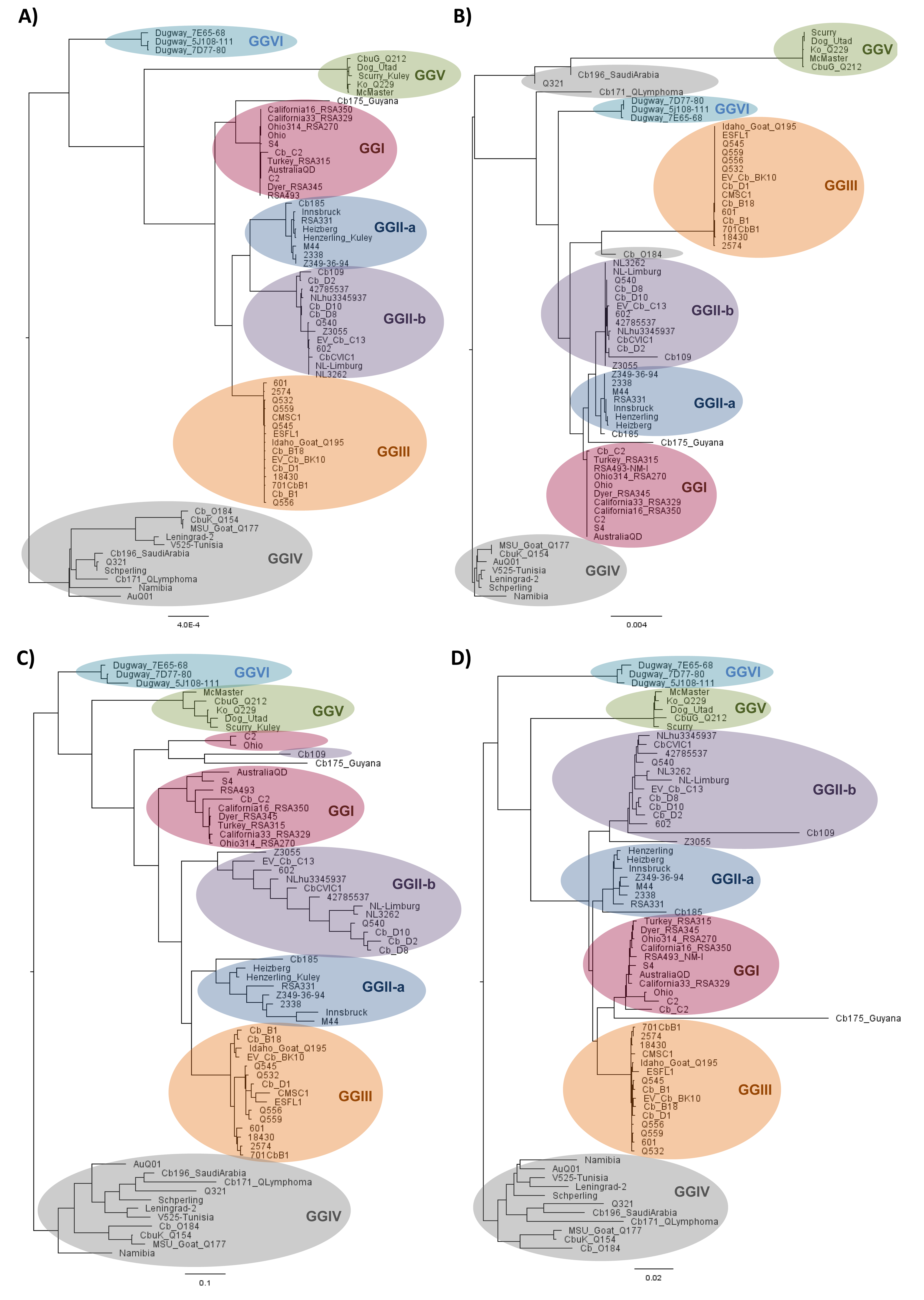

Supplement: Supplementary file 10 — Figure S6. Phylogenetic relationship of 67 C. burnetii isolates based on partial genome content. A) Core genome-based tree determined by Roary, B) Core genome-based tree determined by BPGA, C) Accessory (binary) genome-based tree determined by Roary, and D) Pan genome-based tree determined by BPGA. Newick outputs of both BPGA and Roary pipelines were used to draw trees using FigTree, and Genomic Groups were color coded. All trees were rooted along the branch leading to GG IV (see Methods). (TIF 3891 kb) [file 12864_2019_5833_MOESM10_ESM.tif]

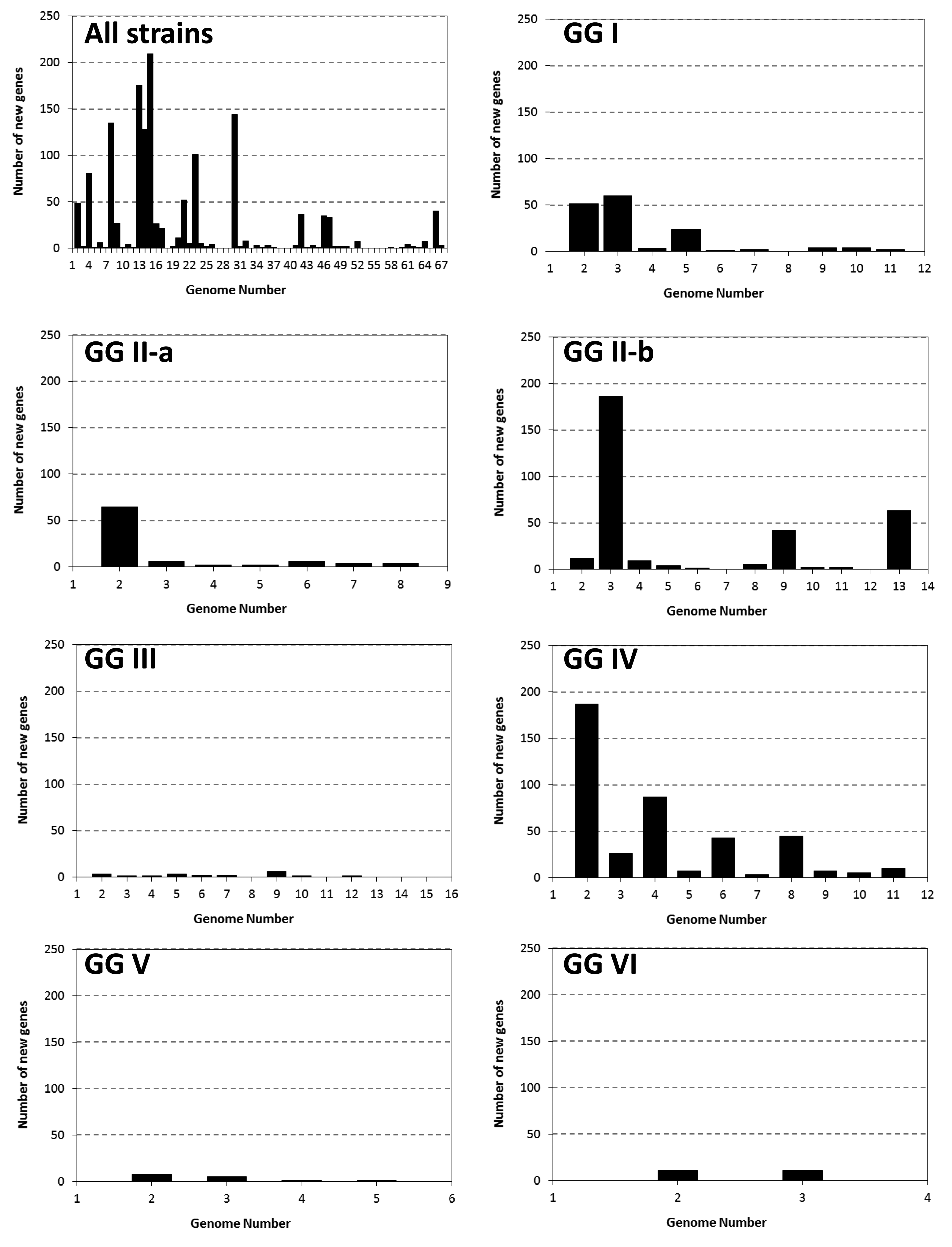

Supplement: Supplementary file 12 — Figure S7. New gene plots after BPGA pan-genome subset analysis using groups of isolates according to their genomic group associations. Proteins annotated using PROKKA were used as input files. The protein similarity threshold for protein clustering was 90%. Genomic groups were assigned as seen in Fig. 1. Note that “new” genes represent polymorphic variants due to the presence of SNPs in existing genes rather than newly acquired genes. (TIF 404 kb) [file 12864_2019_5833_MOESM12_ESM.tif]
