# Supplementary material for: Extensive genome analysis of Coxiella burnetii reveals limited evolution within genomic groups
Source: BMC Genomics. 2019 Jun 5;20:441. doi: 10.1186/s12864-019-5833-8 (PMC6549354; doi:10.1186/s12864-019-5833-8)
Supplement: Supplementary file 11 — Table S4. Results of a PANTHER gene enrichment analysis of core and accessory genome contents of 67 C. burnetii isolates. Note that no significant enrichment was found in the unique genome. (PDF 29 kb) [file 12864_2019_5833_MOESM11_ESM.pdf]

Table S4: Results of a PANTHER gene enrichment analysis of core and accessory genome contents of 67 *C. burnetii* isolates. Results for PANTHER GO Slim Biological Process, GO Slim Molecular Function, and Cellular Component Complete are reported. In all cases: Bonferroni correction = true; Bonferroni count = 83. See Methods for details.

**Core _GO-Slim Biological Process**	*C. burnetii* - reference (n=1812)	Core_genome (n=1194)	Core_genome (expected)	fold Enrichment	over / under (+ / -)	P-value
primary metabolic process	380	312	250.40	1.25	+	1.07E-03
metabolic process	476	392	313.66	1.25	+	2.61E-05
cellular process	406	325	267.53	1.21	+	5.06E-03
**Accessory _GO-Slim Biological Process**	*C. burnetii* - reference (n=1812)	Access._genome (n=498)	Access._genome (expected)	fold Enrichment	over / under (+ / -)	P-value
metabolic process	476	97	130.56	0.74	-	2.45E-02
**Core _ GO-Slim Molecular Function**	*C. burnetii* - reference (n=1812)	Core_genome (n=1194)	Core_genome (expected)	fold Enrichment	over / under (+ / -)	P-value
catalytic activity	811	667	534.40	1.25	+	2.92E-12
**Accessory _ GO-Slim Molecular Function**	*C. burnetii* - reference (n=1812)	Access._genome (n=498)	Access._genome (expected)	fold Enrichment	over / under (+ / -)	P-value
catalytic activity	811	182	222.44	0.82	-	4.46E-02
**Core _ GO Cellular Component**	*C. burnetii* - reference (n=1812)	Core_genome (n=1194)	Core_genome (expected)	fold Enrichment	over / under (+ / -)	P-value
cytoplasm	404	327	266.21	1.23	+	1.18E-03
intracellular part	432	352	284.66	1.24	+	2.57E-04
intracellular	459	374	302.45	1.24	+	1.05E-04
cell part	608	492	400.64	1.23	+	1.17E-06
cell	614	496	404.59	1.23	+	1.22E-06
**Accessory _ GO Cellular Component**	*C. burnetii* - reference (n=1812)	Access._genome (n=498)	Access._genome (expected)	fold Enrichment	over / under (+ / -)	P-value
cytoplasm	404	81	110.81	0.73	-	3.09E-02
intracellular part	432	84	118.49	0.71	-	5.90E-03
intracellular	459	91	125.90	0.72	-	6.98E-03
